# Kruppel-like factor 2 contributes to blood-spinal cord barrier integrity and functional recovery from spinal cord injury by augmenting autophagic flux

**DOI:** 10.7150/thno.74324

**Published:** 2023-01-01

**Authors:** Zili He, Jiqing Du, Yu Zhang, Yitie Xu, Qian Huang, Qingwei Zhou, Min Wu, Yao Li, Xie Zhang, Hongyu Zhang, Yuepiao Cai, Keyong Ye, Xiangyang Wang, Yingze Zhang, Qi Han, Jian Xiao

**Affiliations:** 1 Oujiang Laboratory (Zhejiang Lab for Regenerative Medicine, Vision and Brain Health), Department of Orthopaedics Surgery, The Second Affiliated Hospital and Yuying Children's Hospital of Wenzhou Medical University, Wenzhou, 325035, China.; 2 Department of Orthopaedics, Affiliated Pingyang Hospital and School of Pharmaceutical Science, Wenzhou Medical University, Wenzhou, 325000, China.; 3 Department of Pharmacy, Ningbo Medical Treatment Center Li Huili Hospital, Ningbo, 315040, China.; 4 Department of Orthopaedic Surgery, The Third Hospital of Hebei Medical University, Shijiazhuang, 050051 China.; 5 Department of Anatomy and Physiology, Shanghai Jiao Tong University School of Medicine, Shanghai, 200025, China.

**Keywords:** Blood-spinal cord barrier, Tight junction proteins, Spinal cord injury, Kruppel-like factor 2, Autophagy-lysosomal pathway

## Abstract

**Background:** Increasing evidence suggests that acute traumatic spinal cord injury (SCI)-induced defects in autophagy and autophagy-lysosomal pathway (ALP) may contribute to endothelial barrier disruption following injury. Recently, Kruppel-like factor 2 (KLF2) was reported as a key molecular switch on regulating autophagy. Whether KLF2 coordinates endothelial endothelial ALP in SCI is not known.

**Methods:** Genetic manipulations of KLF2 were performed in bEnd.3 cells and SCI model. Western blot, qRT-PCR, immunofluorescence staining and Lyso-Tracker Red staining, Evans blue dye extravasation, behavioral assessment via Basso mouse scale (BMS), electrophysiology and footprint analysis were performed.

**Results:** In SCI, autophagy flux disruption in endothelial cells contributes to TJ proteins degradation, leading to blood-spinal cord barrier (BSCB) impairment. Furthermore, the KLF2 level was decreased in SCI, overexpression of which alleviated TJ proteins loss and BSCB damage, which improve motor function recovery in SCI mice, while knockdown of KLF2 displayed the opposite effects. At the molecular level, KLF2 overexpression alleviated the TJ proteins degradation and the endothelial permeability by tuning the ALP dysfunction caused by SCI and oxygen glucose deprivation (OGD).

**Conclusions:** Endothelial KLF2 as one of the key contributors to SCI-mediated ALP dysfunction and BSCB disruption. KLF2 could be a promising pharmacological target for the management and treatment of SCI.

## Introduction

The barrier mechanisms, including blood-brain barrier (BBB) and blood-spinal cord barrier (BSCB), at the blood central nervous system (CNS) interface are morphologically and functionally uniform along the entire neuraxis. Similar to BBB, the BSCB directs molecular exchanges between the circulating blood and the blood and spinal cord to maintain CNS homeostasis 1. Structurally, BSCB is formed by the self-fusion of endothelial cells with tight junction (TJ) proteins and by interactions with astrocytes, pericytes and perivascular microglia 2. Under traumatic spinal cord injury (SCI), the lesion-induced hypoxic microenvironment could reduce the expression of TJ proteins and induce endothelial cell dysfunction and promote BSCB disruption. Accumulating evidence indicates that BSCB disruption contributes to inflammation and ischemia, leading to the progression of spinal nervous tissue damage 3. Therefore, modulating the BSCB function may significantly improve motor function following SCI.

Autophagy is a lysosome-dependent catabolic pathway that eliminates many misfolded proteins and cellular organelles to maintain homeostasis 4. Our previous studies revealed that activation of autophagy attenuated BSCB damage after SCI 5, 6. Recent research indicates that autophagy protects BBB integrity by inhibiting the redistribution of membranous claudin-5 (a member of TJ proteins) and participating in the degradation of claudin-5 in the cytosol 7. Autophagy flux disruption in SCI due to lysosomal dysfunction has been shown to induce endoplasmic reticulum stress and apoptosis in neurons 8, 9. However, the significant contribution of autophagy-lysosomal pathway (ALP) dysfunction in endothelial cells after SCI remains indefinite. Moreover, lysosomal dysfunction perturbs autophagic clearance, accumulating mitochondrial dysfunction and the induction of mitochondrial oxidative stress, resulting in abnormal tight junctions and epithelial dysfunction 10. Furthermore, autophagy-deficient mice displayed cerebrovascular barrier dysfunction and a loss of TJ proteins 11. Hence, endothelial ALP in SCI plays a significant role in the pathophysiology of BSCB.

Kruppel-like factors (KLFs), the zinc finger family of transcription factors, comprise 17 members, which regulate cell growth and differentiation 12. The Kruppel-like factor 2 (KLF2), abundantly expressed in endothelial cells, was identified as a “molecular switch” for regulating vascular function 13. KLF2 deficiency in primary endothelial cells derived from KLF2(+/-) mice could significantly enhance thrombin-induced endothelial barrier permeability 14. Recent works have revealed that KLF2 overexpression protects against ischemic stroke and alleviates BBB dysfunction by regulating the expression of OCC (occludin, a member of TJ proteins) in endothelial cells 15. Recently, Zhao and colleagues demonstrated that KLF2 enhanced neurological function recovery in rat SCI model, following treatment with miR-125b 16. Nevertheless, the exact cellular mechanisms of KLF2 in regulating BSCB function after SCI have never been explored. Emerging studies have indicated that KLFs regulate autophagy 17-20. The KLF-autophagy pathway modulated the life span of nematodes and regulated mammalian age-associated vascular dysfunction 20. In addition, previous research has indicated that KLF2 modulates autophagy during osteoblast differentiation and osteoclastogenesis 17, 18. At the molecular level, KLFs have been demonstrated to activate autophagy by binding to the promoter region of autophagy genes 17, 18, 21. Our results revealed that the expression of KLF2 in endothelial cells peaked at 1 day and dropped sharply at 3 days after SCI, which was associated with autophagy flux disruption and lysosome dysfunction. Therefore, we speculated that KLF2 contributed to regulating ALP in endothelial cells and maintaining BSCB integrity after SCI. We used the SCI mouse model and an OGD model in bEnd.3 cells. Our findings demonstrated that overexpression of KLF2 alleviated the endothelial ALP dysfunction, inhibited TJ proteins degradation and decreased BSCB permeability to improve functional recovery after SCI. In contrast, the knockdown of KLF2 exerted the opposite effects. Therefore, our findings proposed that modulation of KLF2 activities can deliver a promising therapeutic avenue for the treatment and management of SCI.

## Results

### SCI induces endothelial ALP dysfunction

**Figure 1:**
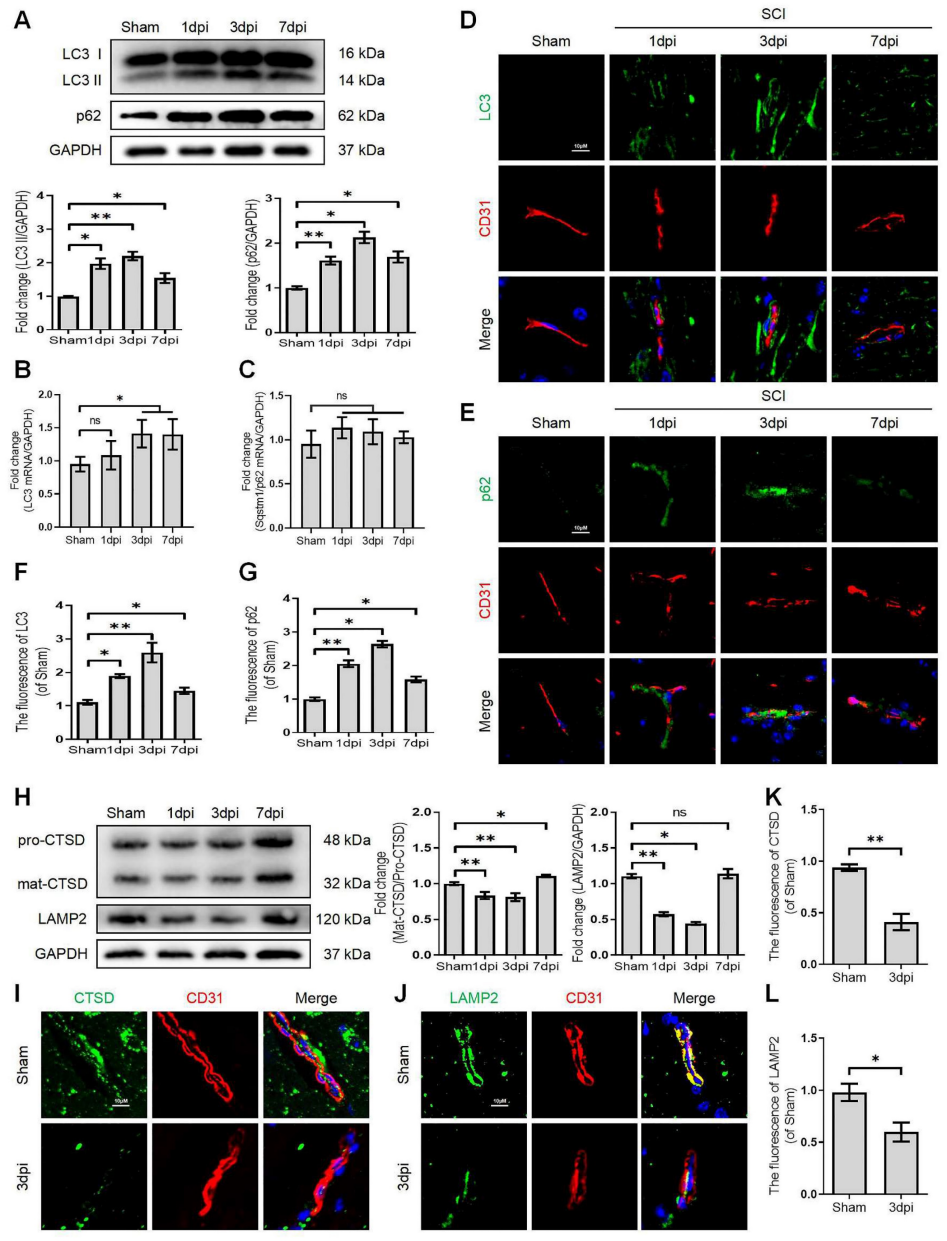
** Disruption of autophagic flux in endothelial cells after SCI.** (A) Western blot and quantification of autophagy markers (LC3 and p62) in spinal cord tissue prepared from Sham and SCI mice at the indicated time points. n = 5 mice in each group. (B-C) Relative mRNA level of LC3 (B) and p62 (C) in the spinal cord prepared from Sham and SCI mice at the indicated time points. n = 5 mice in each group. (D-G) Representative immunofluorescence images and quantification of LC3 (D) and p62 (E) in endothelial cells (marked by CD31) in spinal cord prepared from Sham and SCI (1dpi, 3dpi and 7dpi) mice. n = 5 mice in each group. (H) Western blot and quantification of lysosome markers (CTSD and LAMP1) in spinal cord tissue prepared from Sham and SCI mice at the indicated time points. n = 5 mice in each group. (I-L) Representative immunofluorescence images and quantification of CTSD (I) and LAMP2 (J) in endothelial cells (marked by CD31) in spinal cord prepared from Sham and SCI (3dpi) mice. n = 5 mice in each group. Shown are mean values ± SEM; ns stands for not significant, * P < 0.05, ** P < 0.01.

**Figure 2:**
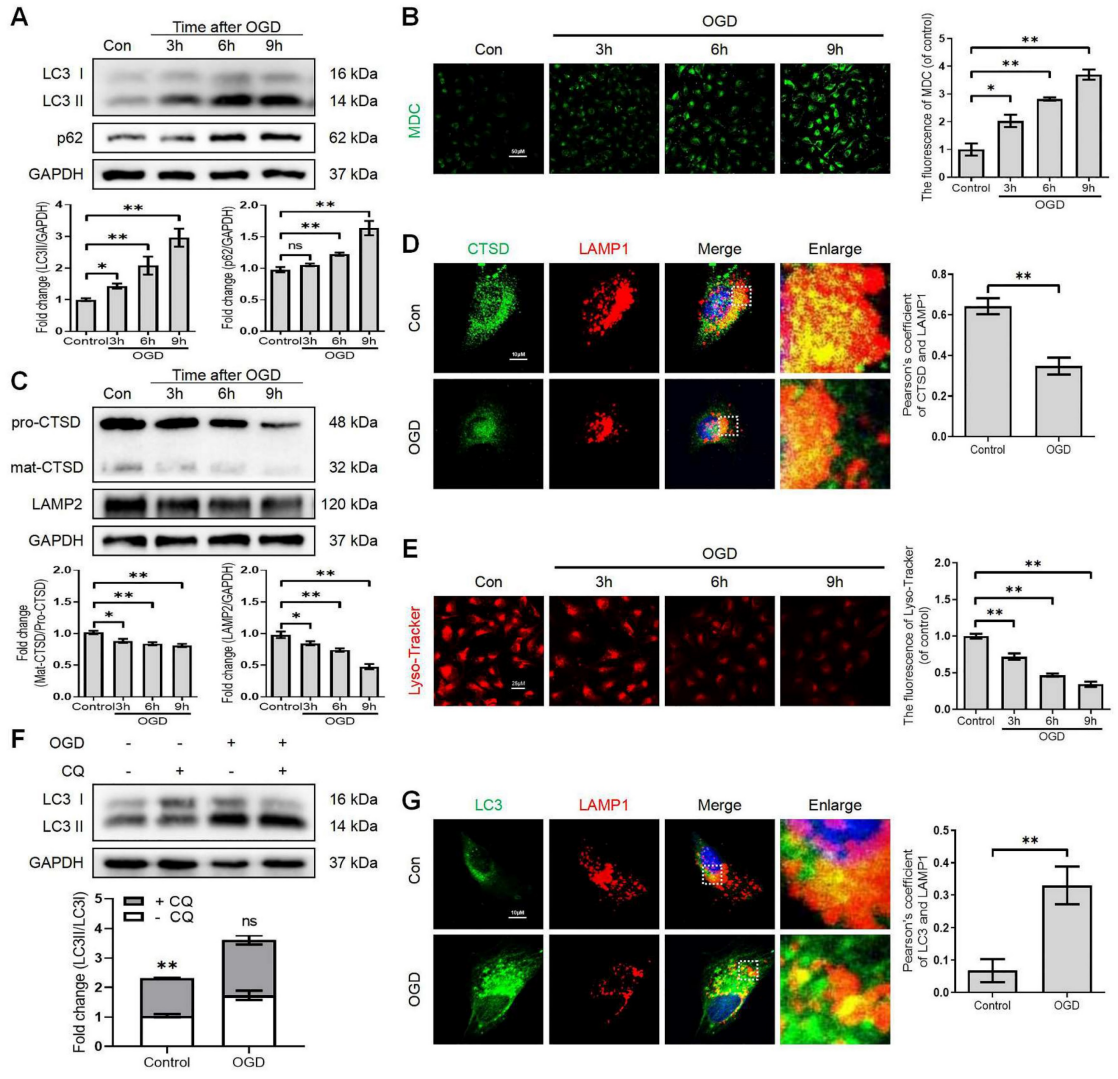
** Lysosome dysfunction in bEnd.3 cells after SCI.** (A) Western blot and quantification of autophagy markers (LC3 and p62) in bEnd.3 cells after OGD treatment for different periods. n = 4 samples. (B) Representative images and quantification analysis of MDC staining in bEnd.3 cells after OGD treatment. n = 3 samples. (C) Western blot and quantification of lysosome markers (CTSD and LAMP2) in bEnd.3 cells after OGD treatment for different periods. n = 4 samples. (D) Representative immunofluorescence images and quantification of CTSD in LAMP1-labeled lysosome after OGD treatment for 9 h. n = 4 sample. (E) Representative images and quantification analysis of Lyso-Tracker staining in bEnd.3 cells after OGD treatment. n = 3 samples. (F) Effect of OGD (9 h) on protein expression of LC3 from bEnd.3 cells pretreated in the absence or presence of CQ. n = 3 sample. (G) Representative immunofluorescence images and quantification of LC3 in LAMP1-labeled lysosome after OGD treatment for 9 h. n = 4 sample. Shown are mean values ± SEM; ns stands for not significant, * P < 0.05, ** P < 0.01.

Growing evidence suggests that autophagy maintains the integrity of microvascular barriers 22. Our previous study demonstrated that SCI-mediated neuronal autophagic-flux disruption could induce neuronal death and impede neurological repair following injury 9. However, the promising role and underlying molecular mechanisms of autophagy in endothelial cells after SCI remain elusive. We performed the western blot assay to determine the expression levels of autophagosome protein (LC3) and autophagy substrate protein (p62) after SCI. More importantly, our results revealed that the expression level of LC3-II and p62 proteins were significantly augmented after SCI (Figure 1A). In addition, q-RT-PCR analyses demonstrated that the mRNA level of LC3 was also raised at 1, 3, 7 days after SCI (Figure 1B). However, the mRNA level of p62 was not elevated after SCI (Figure 1C). Immunofluorescence analysis of endothelial cells (marked with CD31) from the lesion site of mice at 1, 3, and 7 days after SCI revealed higher levels of LC3 (Figure 1D and F) and 62 (Figure 1E and G) signals compared to the sham group. To further investigate the disruption of autophagic flux in endothelial cells, we treated OGD to mimic the ischemic damage of endothelial cells after SCI (the appropriate OGD time was determined through a CCK-8 experiment, Figure S1A). Western blot analysis confirmed that the protein expression levels of LC3-II and p62 gradually peaked from 3h to 9 h in OGD-treated bEnd.3 cells (Figure 2A). Furthermore, monodansylcadaverine (MDC) staining for autophagic vacuoles revealed accumulated autophagosomes in the cytoplasm of bEnd.3 cells after OGD treatment (Figure 2B). Taken together, our results indicated that the autophagic flux was disrupted in endothelial cells after SCI.

It has been reported that the lysosome is an essential organelle for executing autophagic activity 23. We next determined the expression of lysosomal-associated membrane protein 2 (LAMP2) and the lysosomal proteases cathepsin D (CTSD) in endothelial cells after SCI. The western blot and immunostaining analyses revealed that the protein expression of LAMP2 and the ratio of mat-CTSD/pro-CTSD were markedly decreased at the lesion site at 1, 3 days after injury but upregulated at 7 days (Figure 1H-L). These time-dependent expression patterns induced by OGD were also observed in bEnd.3 cells (Figure 2C). Similarly, the intensity of CTSD was minimized in the LAMP1-labeled lysosomes at 9 h post-OGD (Figure 2D). In addition, a gradual decrease of red fluorescence of Lyso-Tracker (a sensitive lysosomotropic pH probes) in the bEnd.3 cells were observed, suggesting that OGD stress impaired lysosome function (Figure 2E). Collectively, these findings revealed that the number and function of lysosomes in endothelial cells were impaired at the early phase of SCI.

To further clarify the change of autophagic flux, the LC3 turnover was analyzed in OGD-treated bEnd.3 cells in the absence or presence of chloroquine (CQ, a classic autophagy inhibitor that blocks autophagosome binding to lysosomes) 24. We found that CQ treatment markedly increased LC3Ⅱ level in the control group compared to OGD-treated bEnd.3 cells (Figure 2F). Immunofluorescence analysis indicated increased LC3 signals, decreased LAMP1 expression and low co-localization of LC3 with LAM P1 in bEnd.3 cells at 9 h after OGD (Figure 2G). In summary, these findings indicate that ALP dysfunction in endothelial cells contributes to SCI.

### Disruption of autophagy exacerbates BSCB integrity impairment

**Figure 3:**
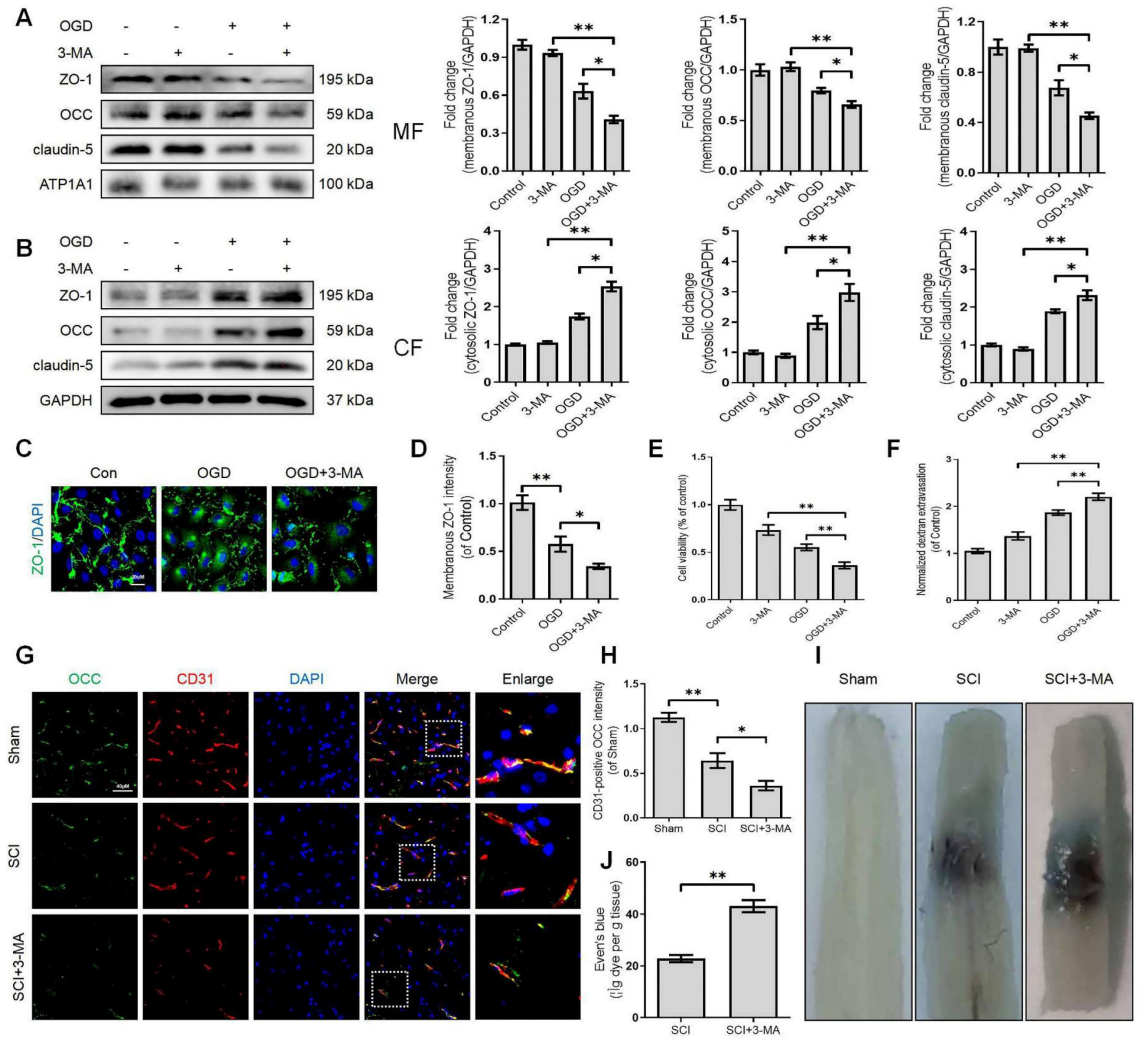
** Disruption of autophagy exacerbates BSCB integrity impairment.** Western blot and quantification of the membranous (A), cytosolic (B) TJs markers (ZO-1, OCC and claudin-5) in bEnd.3 cells after OGD treatment for 9 h in the absence or presence of 3-MA. n = 4 sample. (C-D) Representative immunofluorescence images (C) and quantification (D) of ZO-1. n = 4 sample. (E-F) Cell viability (E) and FITC-dextran extravasation (F) in bEnd.3 cells after of OGD treatment (9 h) in the absence or presence of 3-MA. n = 4. (G-H) Representative immunofluorescence images (G) and quantification (H) of OCC in endothelial cells (marked by CD31). n = 5 mice in each group. (I-J) Representative whole spinal cords of EB extravasation (I) and quantification of EB fluorescence (J) in Sham, SCI (3dpi) and SCI+3-MA group. n = 5 mice in each group. Shown are mean values ± SEM; ns stands for not significant, * P < 0.05, ** P < 0.01.

Growing evidence suggests that autophagy regulates BBB/BSCB integrity. We further explored whether the disruption of autophagy exacerbates the impairment of BSCB integrity after SCI. We administered 3-methyladenine (3-MA), which inhibits autophagy by blocking autophagosome formation via targeting type III phosphatidylinositol 3-kinases (PI-3K). More specifically, we found that 3-MA treatment restored the expression levels of LC3-II protein and increased the expression levels of the p62 protein in OGD-induced bEnd (Figure S1B, D, E and F). Furthermore, 3-MA mediated autophagy inhibition in OGD-induced bEnd.3 cells led to severer delocalization of membranous TJ proteins including ZO-1, OCC and claudin-5 (Figure 3A) and accumulation of cytosolic ZO-1, OCC and claudin-5 (Figure 3B). Similarly, OGD treatment could reduce the intensity of membranous ZO-1 in bEnd.3 cells, which was exacerbated by 3-MA administration (Figure 3C-D). We also checked the correlation between autophagy and endothelial cell apoptosis. The results demonstrated that inhibition of autophagy minimized cell viability (Figure 3E) and induced apoptosis levels (Figure S1C, D and G). Notably, inhibition of autophagy intensified the infiltration of FITC-dextran through the endothelial monolayer with 3-MA treatment compared to the OGD groups (Figure 3F). Based on these in-vitro findings, we explored whether autophagy inhibition could disrupt BSCB permeability following SCI. Our results indicated that intraperitoneal injection of 3-MA into SCI mice (Figure S2A-C) could exacerbate the loss of TJ proteins (Figure 3G-H) and Evans blue (EB) dye extravasation (Figure 3I-J). Collectively, these findings reveal that the disruption of autophagy exacerbates the impairment of BSCB integrity after SCI.

### KLF2 responds to SCI

**Figure 4:**
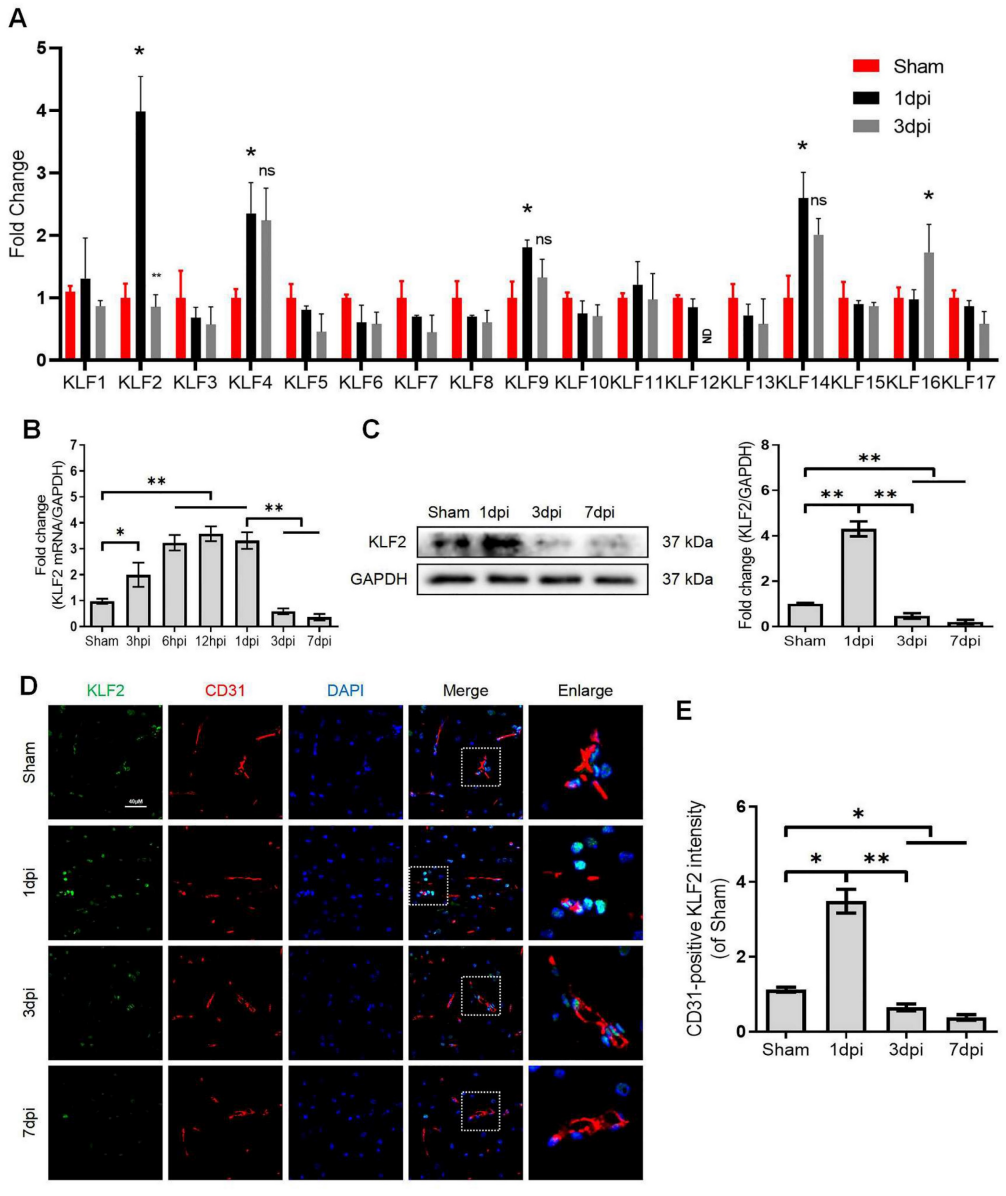
** KLF2 expression after SCI.** (A) Relative mRNA level of 17 KLF family members in the spinal cord prepared from Sham and SCI mice at the indicated time points. n = 3 mice in each group. (B-C) Expression of mRNA encoding KLF2 (B) and protein expression of KLF2 (C) in the spinal cord prepared from Sham and SCI mice at the indicated time points. n = 5 mice in each group. (D) Representative immunofluorescence images and quantification of KLF2 in endothelial cells (marked by CD31) in spinal cord prepared from Sham and SCI (3dpi) mice. n = 5 mice in each group. Shown are mean values ± SEM; ns stands for not significant, * P < 0.05, ** P < 0.01.

KLFs are a family of transcription factors that regulate integral functions of endothelial cells including cell proliferation, differentiation, growth and migration 25. Recent studies indicated that autophagy could be a key molecular mechanism of KLFs in regulating endothelial homeostasis and barrier integrity 20, 21, 26. However, the contributory roles of KLFs in maintaining endothelial barrier function by modulating autophagy in SCI are not well-characterized. Herein, we unbiasedly evaluated the mRNA expression of 17 KLFs (KLF1, KLF2, KLF3, KLF4, KLF5, KLF6, KLF7, KLF8, KLF9, KLF10, KLF11, KLF12, KLF13, KLF14, KLF15, KLF16 and KLF17) in the sample of spinal lesion center at 1 and 3 days after SCI. Our results indicated that the mRNA expression of KLF2, KLF4, KLF9 and KLF14 levels were significantly upregulated at 1 day after SCI. Intriguingly, KLF2 showed a sharp downregulation at 3 days after injury (Figure 4A). Of note, the remarkable drop of KLF2 mRNA levels at 3 days after SCI coincided with the autophagosomes accumulation and lysosomal dysfunction. To further validate these findings, we detected the mRNA levels of KLF2 over an extended period in SCI. The results demonstrated that expression levels of KLF2 mRNA gradually elevated from 3h to 1d after SCI, followed by a sharp drop at 3 and 7 days (Figure 4B). Western blot analysis of KLF2 protein expression also revealed the same tendencies (Figure 4C). Similarly, the fluorescence intensity of KLF2 in endothelial cells of the lesion site also peaked at 1day after injury, gradually decreasing by 3 and 7 days (Figure 4D-E). Meanwhile, in-vitro assays also revealed the downregulation of KLF2 protein in OGD-induced bEnd.3 cells (Figure S3A-B). Collectively, these results suggest that KLF2 deficiency may be associated with ALP in endothelial cells after SCI.

### KLF2 regulates the ALP function in bEnd.3 cells

**Figure 5:**
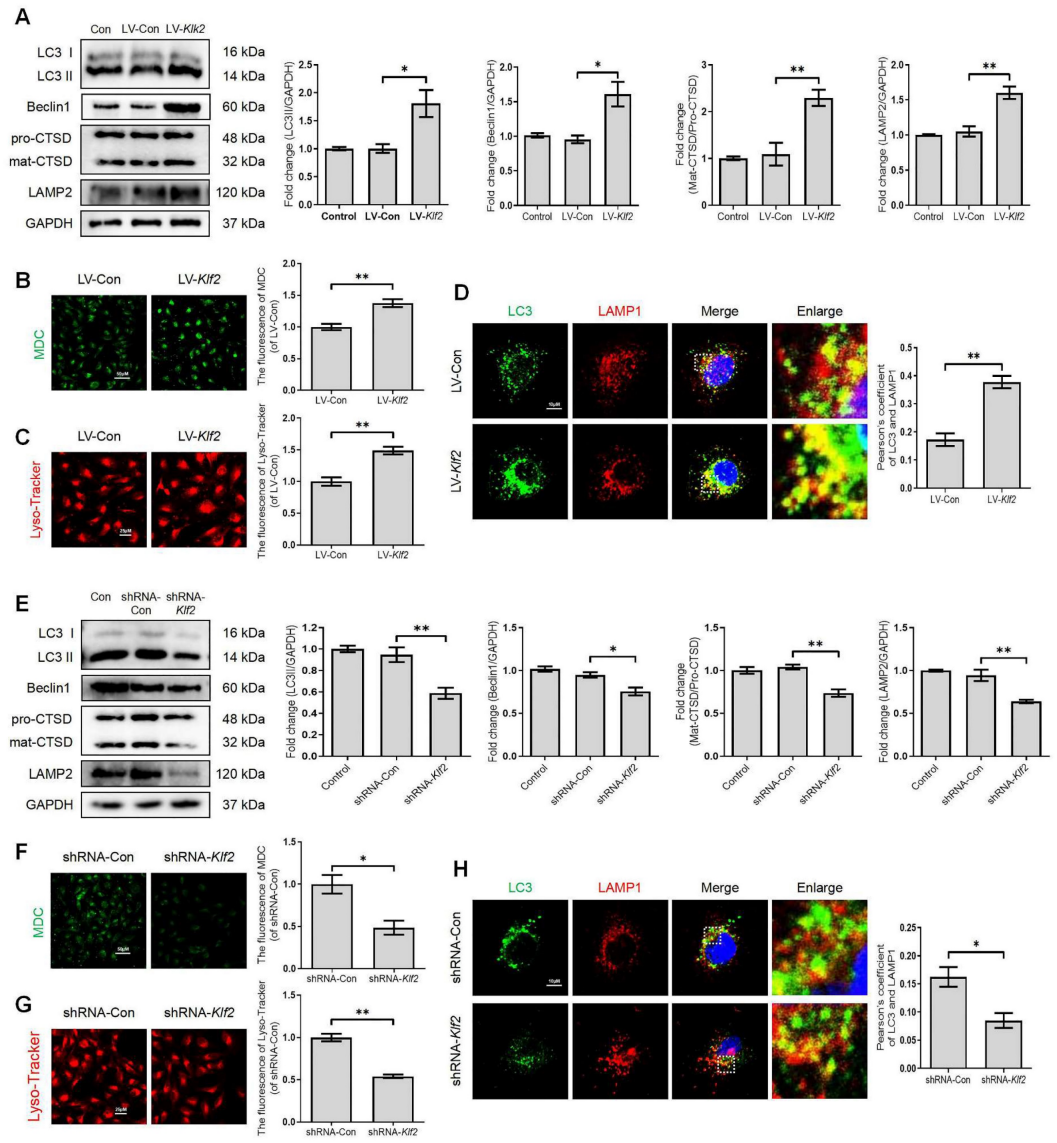
** KLF2 enhances the autophagy lysosome pathway in bEnd.3 cells.** bEnd.3 cells were respectively transfected with lentivirus-Klf2 or lentivirus-Klf2 shRNA to overexpress or knockdown KLF2 expression. (A) Western blot and quantification of autophagy lysosome pathway markers (LC3, p62, CTSD and LAMP2) in bEnd.3 cells transfected with lentivirus-Klf2. n = 4-5 samples. (B-C) Representative images and quantification of MDC (B) and Lyso-Tracker (C) staining in bEnd.3 cells transfected with lentivirus-Klf2. n = 3 samples. (D) Representative immunofluorescence images and quantification of LC3 and LAMP1 in bEnd.3 cells transfected with lentivirus-Klf2. n = 4 samples. (E) Western blot and quantification of autophagy lysosome pathway markers (LC3, p62, CTSD and LAMP2) in bEnd.3 cells transfected with lentivirus-Klf2 shRNA. n = 4-5 samples. (F-G) Representative images and quantification of MDC (F) and Lyso-Tracker (G) staining in bEnd.3 cells transfected with lentivirus-Klf2 shRNA. n = 3 samples. (H) Representative immunofluorescence images and quantification of LC3 and LAMP1 in bEnd.3 cells transfected with lentivirus-Klf2 shRNA. n = 4 samples. Shown are mean values ± SEM; ns stands for not significant, * P < 0.05, ** P < 0.01.

To further investigate the significant role of KLF2 in regulating the ALP of endothelial cells, we manipulated KLF2 expression in bEnd.3 cells by transfection with either LV-Klf2 (upregulation, Figure S4A) or shRNA-Klf2 (downregulation, Figure S4B). We found that overexpression of KLF2 in bEnd.3 cells with LV-Klf2 could upregulate the expression level of LC3-II, Beclin1 and LAMP2 proteins and promote the ratio of mat-CTSD/pro-CTSD (Figure 5A), accumulation of MDC in autophagic vesicles (Figure 5B) and Lyso-Tracker labeled lysosomes (Figure 5C) as compared to shRNA-Klf2 treated control cells. However, our results revealed that the expression level of p62 was increased in LV-Klf2-treated cells. The difference was not statistically significant (Figure S4C). Similarly, the LV-Klf2-treated bEnd.3 cells displayed extensive colocalization of LC3 with LAMP1, suggesting that overexpression of KLF2 enhanced autophagosome-lysosome fusion (Figure 5D). Likewise, KLF2 knockdown could downregulate the expression level of LC3-II, Beclin1, LAMP2 and CTSD (Figure 5E); while did not affect p62 expression (Figure S4D). Moreover, a lower autophagic vesicles accumulation (Figure 5F), fewer Lyso-Tracker labeled lysosomes (Figure 5G) was observed. Reduced immunofluorescence colocalization of LC3 and LAMP1 (Figure 5H) were also found in bEnd.3 cells with shRNA-Klf2. Taken together, these findings indicate that KLF2 regulates autophagy and autophagosome-lysosome fusion.

### KLF2 overexpression alleviates injury-induced ALP dysfunction

**Figure 6:**
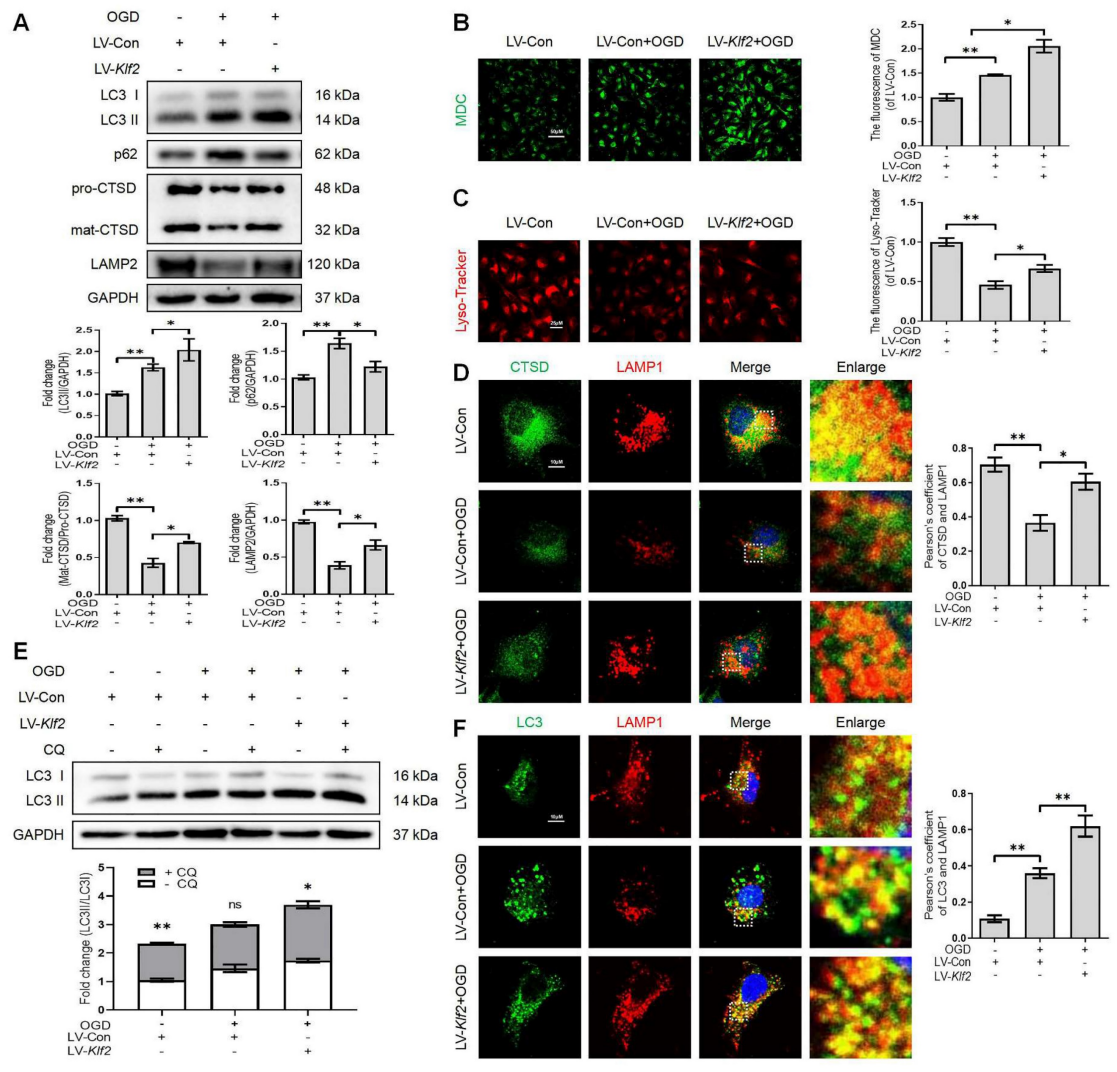
** OGD-induced autophagic flux blockage and lysosome dysfunction is alleviated by KLF2 overexpression in bEnd.3 cells.** (A) Western blot and quantification of autophagy lysosome pathway markers (LC3, p62, CTSD and LAMP2) in bEnd.3 cells respectively transfected with LV-Con or LV-Klf2 after OGD treatment (9 h). n = 3-5 samples. (B-C) Representative images and quantification of MDC staining (B) and Lyso-Tracker staining (C) in bEnd.3 cells. n = 3 samples. (D) Representative immunofluorescence images and quantification of CTSD in LAMP1-labeled lysosome in bEnd.3 cells. n = 4. (E) Effect of OGD (9 h) on protein expression of LC3 from bEnd.3 cells respectively transfected with LV-Con or LV-Klf2, and pretreated in the absence or presence of CQ. n = 3 sample. (F) Representative immunofluorescence images and quantification of LC3 (F) in LAMP1-labeled lysosome in bEnd.3 cells. n = 4. Shown are mean values ± SEM; ns stands for not significant, * P < 0.05, ** P < 0.01.

OGD facilitates autophagic flux disruption and induces lysosome dysfunction (Figure 2). We upregulated KLF2 level in OGD-treated bEnd.3 cells (Figure S5A). Our results demonstrated that KLF2 overexpression reversed the expression level of LC3-II, p62, mat-CTSD/pro-CTSD and LAMP2 proteins (Figure 6A) in OGD-induced bEnd.3 cells. In addition, KLF2 overexpression in OGD-treated bEnd.3 cells increased the number of autophagic vesicles labeled by the MDC staining (Figure 6B) and enhanced lysosomal acidification (Figure 6C), compared with OGD-treated cells expressing non-activating KLF2 sequence. Similarly, bEnd.3 cells exposed to OGD exhibited low intensity of CTSD and LAMP1 puncta. At the same time, KLF2 overexpression increased both CTSD and LAMP1 intensity levels in OGD-treated cells (Figure 6D). The LC3 turnover assay showed that autophagic flux disruption was attenuated by KLF2 overexpression via enhancing LC3 II ratio in OGD and LV-Klf2 treated bEnd.3 cells upon CQ treatment (Figure 6E). We also found that overexpression of KLF2 increased the colocalization of LC3 and LAMP1, while they remained relatively distinct in bEnd.3 cells exposed to OGD infected with control lentivirus (Figure 6F). In summary, these results suggest that overexpression of KLF2 ameliorates OGD-induced ALP dysfunction in bEnd.3 cells.

**Figure 7:**
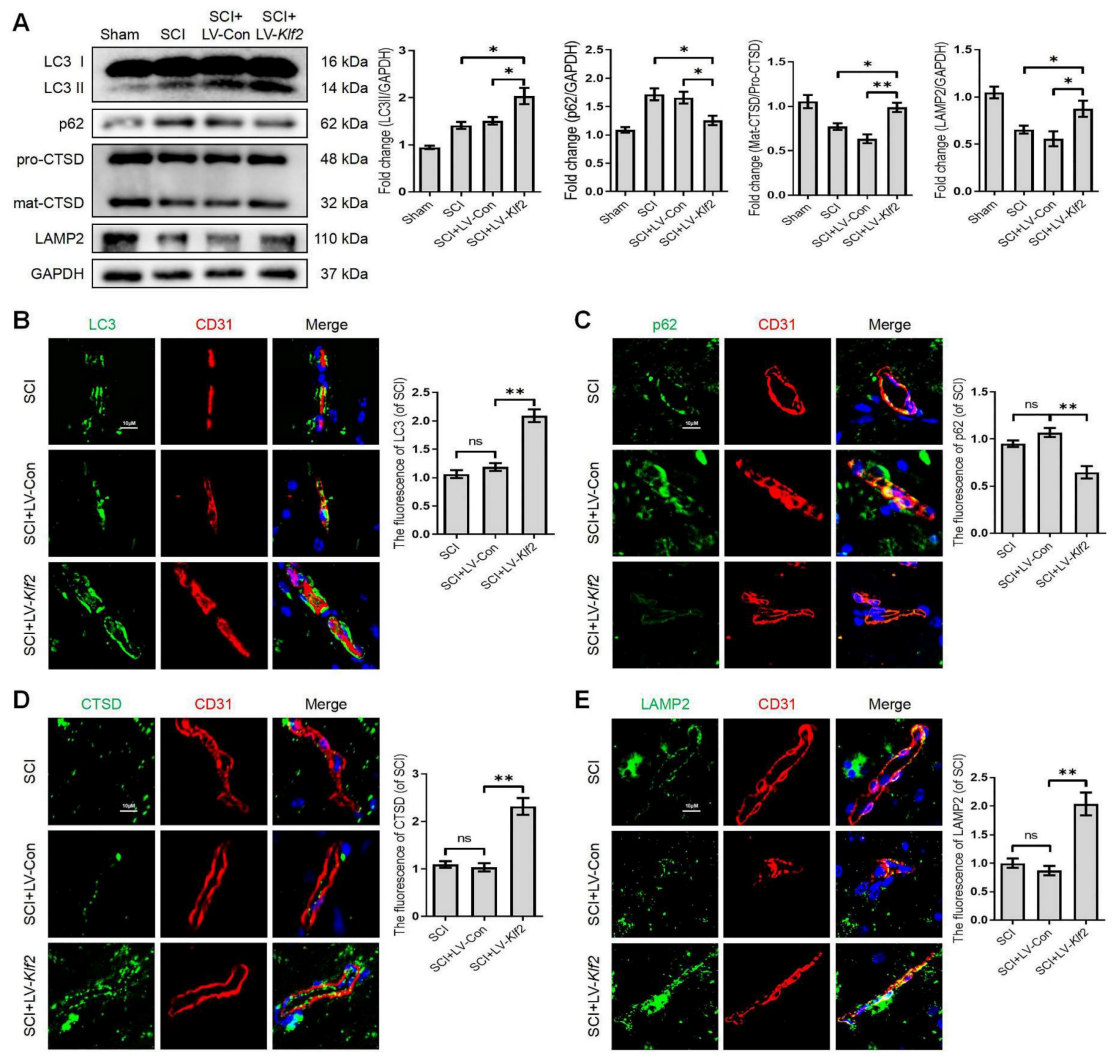
** Overexpression KLF2 improves autophagic flux blockage and lysosome dysfunction after SCI.** (A) Western blot and quantification of autophagy lysosome pathway markers (LC3, p62, CTSD and LAMP2) in spinal cord tissue prepared from Sham, SCI mice, and mice injected with LV-Con or LV-Klf2, then subjected with SCI, at day 3 after injury. n = 5 mice in each group. (B-E) Representative immunofluorescence images and quantification of LC3 (B), P62 (C), CTSD (D) and LAMP2 (E) in endothelial cells (marked by CD31) in indicated groups at day 3 after injury. n = 5 mice in each group. Shown are mean values ± SEM; ns stands for not significant, * P < 0.05, ** P < 0.01.

To assess whether KLF2 overexpression also plays a similar role in vivo, we used the SCI model and transfected mice with lentiviral vectors (LV-Klf2 and LV-Con) via intrathecal injection at 5 days before SCI (Figure S6A and C). Overexpression of KLF2 enhanced LC3-II expression; while did not modify p62 expression (Figure S6B). The western blot analysis revealed that KLF2 overexpression upregulated the expression level of LC3-II, LAMP2 and mat-CTSD/pro-CTSD proteins and downregulated the expression level of p62 protein in SCI mouse models (Figure 7A). Meanwhile, the fluorescence intensity of LC3, p62, CTSD and LAMP2 in endothelial cells of the lesion site from each group showed same tendencies (Figure 7B-E). Taken together, these findings revealed that overexpression of KLF2 could remarkably alleviate ALP dysfunction in endothelial cells after SCI.

TFEB, as a member of the MiT family, was identified as a master gene for lysosomal biogenesis, which coordinated ALP by regulating the expression of autophagy and lysosomal genes 27. To determine whether TFEB can regulate KLF2-mediated ALP, we silenced TFEB using Ad-shTFEB (Figure S7A) in OGD-induced bEntd.3 cells. TFEB knockdown reversed LV-KLF2-induced upregulation of LC3II and LAMP2 and downregulation of p62 (Figure S7B). To further test whether KLF2 can directly regulate TFEB expression, we overexpressed LV-KLF2 to measure TFEB mRNA and protein expression levels. More importantly, our results revealed that KLF2 overexpression significantly increased TFEB mRNA and protein expression levels (Figure S7C and D). At the same time, the knockdown of KLF2 downregulated TFEB mRNA and protein expression levels (Figure S7E). Immunofluorescence staining showed that the percentage of cells with TFEB-translocated nuclei was also increased in cells transfected with LV-Klf2 (Figure S7F). Taken together, these results reveal that KLF2 overexpression alleviates injury-induced ALP dysfunction.

### KLF2 overexpression restores OGD-induced endothelial dysfunction by modulating autophagy flux

**Figure 8:**
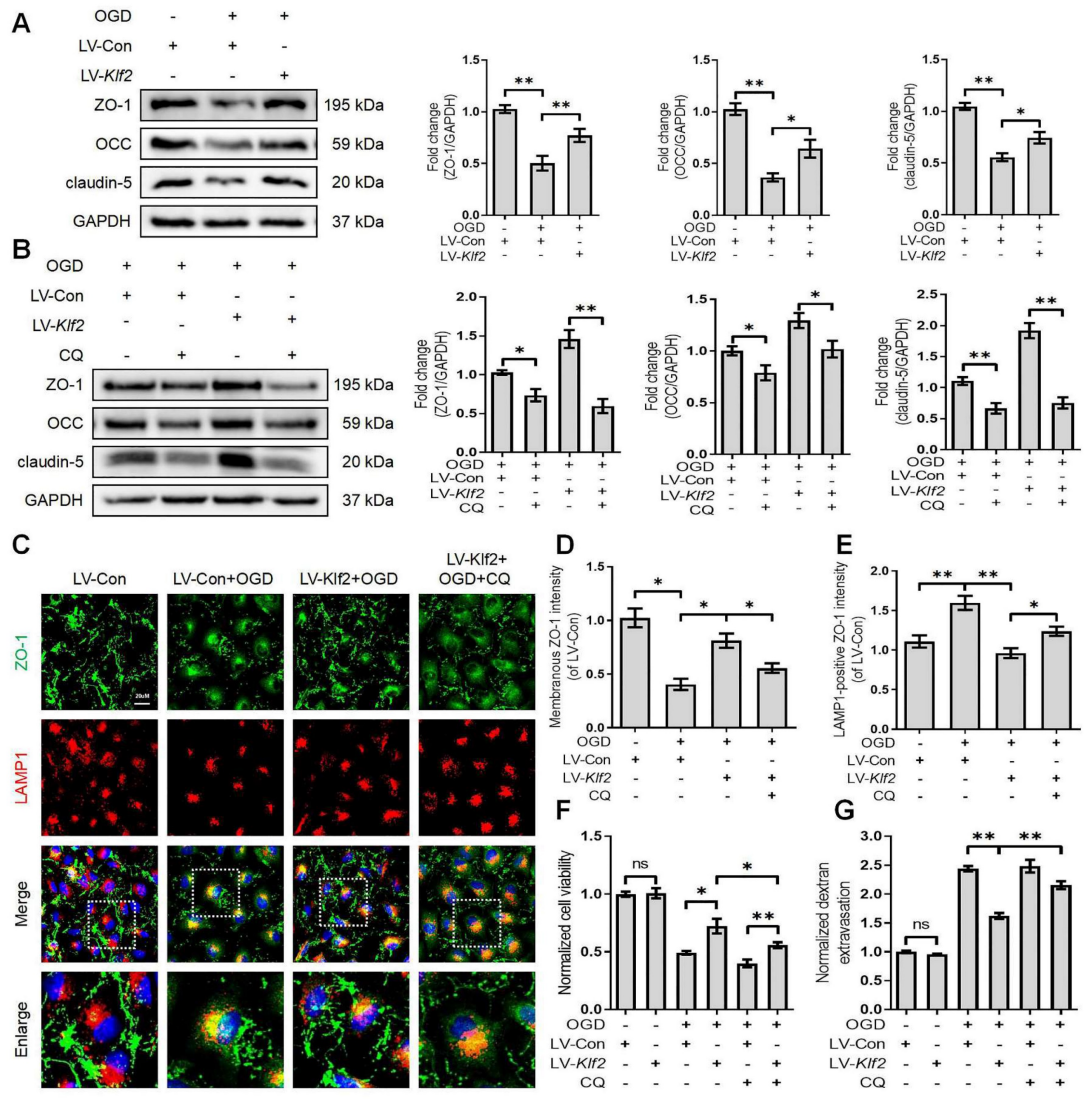
** Overexpression KLF2 improves endothelial barrier via regulating autophagy lysosome pathway in bEnd.3 cells following OGD.** (A) Effect of OGD (9 h) on protein expression of TJs markers (ZO-1, OCC and claudin-5) from bEnd.3 cells respectively transfected with LV-Con or LV-Klf2. n = 4 sample. (B) Effect of OGD (9 h) on protein expression of TJs markers (ZO-1, OCC and claudin-5) from bEnd.3 cells respectively transfected with LV-Con or LV-Klf2, and pretreated in the absence or presence of CQ. n = 4 sample. (C) Representative immunofluorescence images of ZO-1 and LAMP1 in each group. (D-E) Quantification of membranal ZO-1 intensity (D), and LAMP1-positive ZO-1 intensity (E) in each group. n = 4 sample. (F-G) Effect of OGD (9 h) on Cell viability (F) and FITC-dextran extravasation (G) from bEnd.3 cells respectively transfected with LV-Con or LV-Klf2, and pretreated in the absence or presence of CQ. n = 4 sample. Shown are mean values ± SEM; ns stands for not significant, * P < 0.05, ** P < 0.01.

We next explored the underlying mechanism of KLF2 in restoring OGD-induced endothelial dysfunction. We found that overexpression of KLF2 significantly reversed the loss of ZO-1, OCC and claudin-5 in OGD-induced bEnd.3 cells (Figure 8A). The immunofluorescence images also indicated that overexpression of KLF2 partially reduced the loss of membranous ZO-1 and accumulation of LAMP1-positive ZO-1 in the cytosol (Figure 8C, D and E). In addition, KLF2 overexpression remarkably enhanced cell viability (Figure 8F) and suppressed endothelial apoptosis (Figure S5B and D) in OGD-treated bEnd.3 cells. Our results also revealed that KLF2 overexpression reduced the FITC-dextran permeation in OGD-induced bEnd.3 cells (Figure 8G).

Then, we applied CQ to demonstrate whether KLF2 protects endothelial barrier integrity by regulating autophagosome-lysosome fusion after OGD treatment. The blockage of autophagosome-lysosome fusion by CQ treatment abolished the protective effect of KLF2 overexpression on the level of ZO-1, OCC and claudin-5 (Figure 8B), cell viability (Figure 8F), endothelial apoptosis (Figure S5C and D) and ZO-1 abundance in the cell membrane (Figure 8C and D). Furthermore, these changes exacerbated FITC-dextran permeation (Figure 8G). Taken together, these findings reveal that KLF2 overexpression alleviates OGD-induced endothelial dysfunction by modulating autophagy flux in SCI.

### KLF2 improves BSCB integrity via regulating ALP in SCI

**Figure 9:**
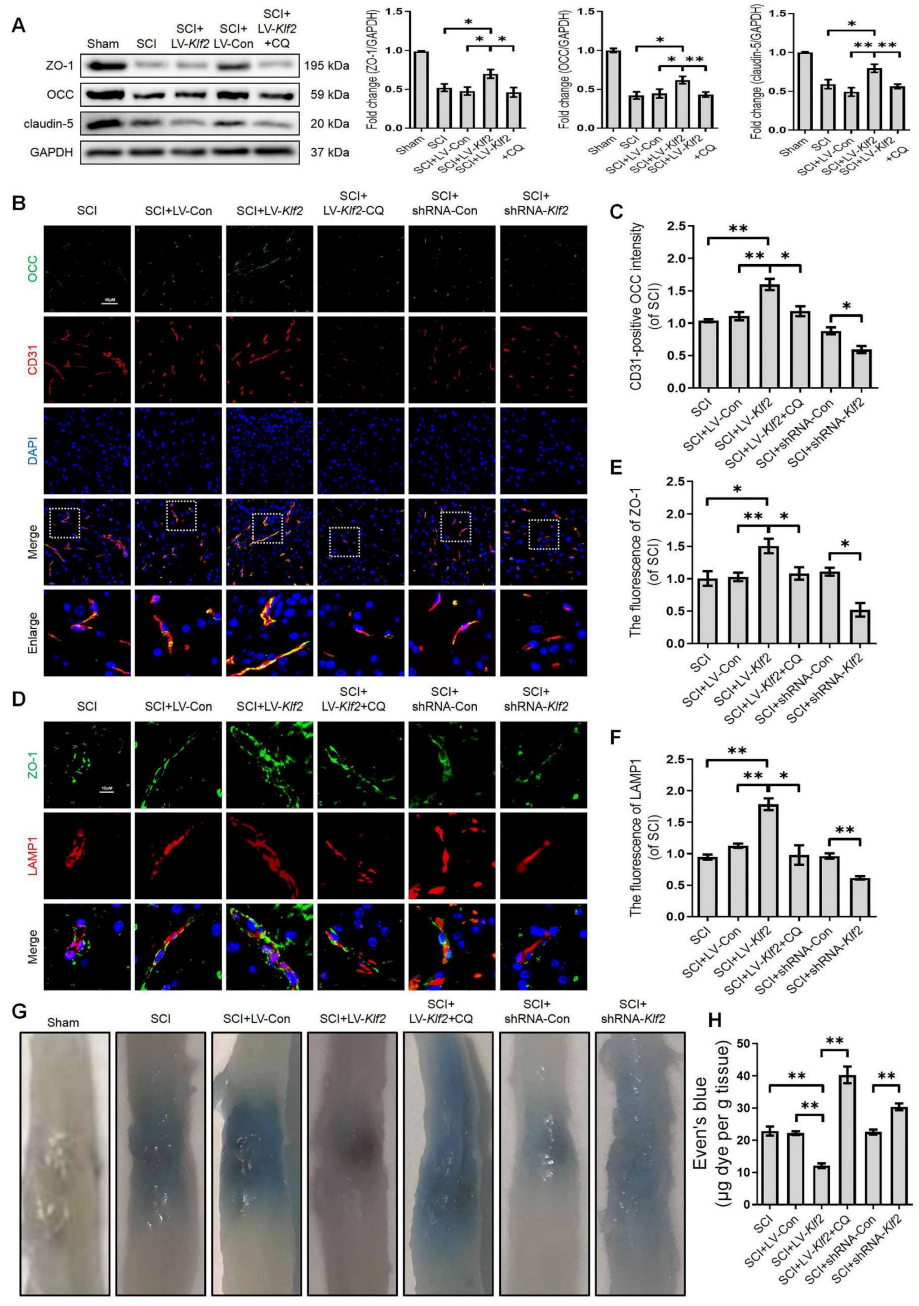
** KLF2 improves BSCB integrity via regulating autophagy lysosome pathway after SCI.** (A) Western blot and quantification of TJs markers (ZO-1, OCC and claudin-5) in spinal cord tissue prepared from Sham, SCI, SCI + LV-Con, SCI + LV-Klf2, and SCI + LV-Klf2 + CQ mice, at day 3 after injury. n = 5 mice in each group. (B-C) Representative immunofluorescence images and quantification of OCC in endothelial cells (marked by CD31) in spinal cord tissue prepared from SCI mice, and mice injected with LV-Con, LV-Klf2, shRNA-Con, shRNA-Klf2 or LV-Klf2 + CQ, then subjected with SCI, at day 3 after injury. n = 5 mice in each group. (D-F) Representative immunofluorescence images and quantification of ZO-1 and LAMP1 in the spinal cord from the indicated groups at day 3 after injury. n = 5 mice in each group. (G-H) Representative whole spinal cords of EB extravasation (G) and quantification of EB fluorescence (H) in the lesions of the indicated groups. n = 5 mice in each group. Shown are mean values ± SEM; ns stands for not significant, * P < 0.05, ** P < 0.01.

To uncover the promising roles of KLF2 on BSCB integrity in vivo, we examined the expression level of TJ proteins (ZO-1, OCC and claudin-5) at 3 days after SCI. Western blot analysis revealed that shRNA-mediated knockdown of KLF2 (Figure S6D) exacerbated the expression level of TJ proteins (ZO-1, OCC and claudin-5) after SCI (Figure S8A). We also found that overexpression of KLF2 (Figure S6C) partially rescued the expression levels of TJ proteins including ZO-1, OCC and claudin-5 after SCI (Figure 9A). Combined with CD31 immunofluorescence staining, the intensity of OCC in CD31-positive endothelial cells was increased or decreased in SCI mice in response to lentivirus-mediated KLF2 upregulation or downregulation, respectively (Figure 9B-C). Furthermore, the overexpression of KLF2 could enhance the fluorescence signal of ZO-1 and LAMP1-labeled lysosomes and the knockdown of KLF2 could markedly minimize the intensity of ZO-1 and LAMP1 (Figure 9D-F). The EB extravasation assay further demonstrated that the knockdown of KLF2 increased EB dye leakage at 3 days after the injury compared with the SCI group and shRNA-Con group, while overexpression of KLF2 attenuated the leakage (Figure 9G-H). In addition, our results revealed that CQ treatment could markedly downregulate the expression level of LV-Klf2 mediated TJ proteins (Figure 9A-F) and inhibit EB dye leakage (Figure 9G and H) in LV-Klf2 transfected mice at 3 days after SCI.

We next explored whether KLF2 played a functional role in restoration following SCI. As shown in Figure S8B and C, SCI induced a dramatic decrease in the amplitude of motor-evoked potentials (MEPs). Replenishing KLF2 in endothelial cells could increase MEP amplitude by about 10% compared to the control group. Intriguingly, enhanced electrophysiological signals were entirely abolished by a subsequent administration of CQ, suggesting that KLF2-mediated neural transmission recovery required lysosomal function (Figure S8B-C). Contrarily, the loss of KLF2 via shRNA-mediated knockdown in SCI mice would further reduce the MEP activity (Figure S8B-C). Moreover, BMS behavioral analysis indicated that overexpression of KLF2 significantly improved locomotor functional scores (Figure S8D) and induced longer stride lengths in the footprint analysis (Figure S8E-F). Intriguingly, improvement in BMS scores and footprints were reversed by CQ treatment, suggesting that KLF2 enhanced motor function recovery after SCI via inhibiting autophagic flux disruption (Figure S8D-F). Conversely, the knockdown of KLF2 in SCI mice showed decreased score of hind-limb locomotor function (Figure S8D) and shorter stride length (Figure S8E-F). Collectively, these findings indicated that promoting autophagy flux could facilitate BSCB protection and improve functional recovery by regulating KLF2 after SCI.

## Discussion

It is well known that autophagy maintains cellular homeostasis via an ALP. Impaired autophagic flux in neurons induces apoptosis and disrupts tissue repair following SCI 8, 9. The underlying mechanism of autophagic flux disruption in endothelial cells and the contributory role of ALP in BCSB integrity and functional recovery after SCI are still unclear. In the present study, we found that both the autophagy marker LC3 and the substrate protein p62 were dramatically increased in OGD-induced bEnd.3 cells and SCI mice, suggesting that autophagy flux was disrupted in endothelial cells. In addition, we found that the expression of lysosomal membrane protein LAMP2 and the proteolytic enzyme CTSD were markedly downregulated and Lyso-Tracker fluorescent signals were reduced, suggesting that lysosomal dysfunction was induced in endothelial cells after SCI. Although previous studies reported that autophagy was mainly activated in neurons 9, 28, our study proved that endothelial cells had profound autophagy impairment following SCI.

The promising role of autophagy in BBB/BSCB is still controversial. The protective and deleterious aspects have been reported in different models in response to various stimuli. Pioneering research revealed that disruption of autophagy flux induced OCC degradation, leading to BBB damage in rat ischemic stroke models 29. Yang et al. showed that hypoxia-induced autophagy activation alleviated BBB disruption by degrading caveolin-1 in the cytoplasm, which inhibited caveolin1-mediated endocytosis of claudin-5. Meanwhile, autophagy was involved in the degradation of intracytoplasmic aggregated claudin-5, thereby reducing cytotoxicity mediated by aggregated proteins in the cytosol and suppressing endothelial cell apoptosis 7.

Interestingly, our studies demonstrated that OGD-induced autophagic flux disruption could not substantially inhibit the delocalization of membranous ZO-1 into the cytosol. Meanwhile, the delocalized membranous proteins including ZO-1 in the cytosol could not eliminate defective autophagy and induce endothelial apoptosis. The loss of membranous ZO-1 and the decrease of endothelial viability in bEnd3 cells treated with OGD may further facilitate the disruption of the endothelial barrier. Besides, our results indicated that pharmacological blockage of autophagy in endothelial cells aggravated the loss of membranous TJ proteins and BSCB disruption after SCI. These results display that autophagy impairment may promote endothelial dysfunction after SCI.

KLFs are a family of transcription factors regulating many cellular processes such as inflammation, proliferation, growth, apoptosis, differentiation, plasticity and migration. Among the family, KLF2, KLF4, KLF6 and KLF15 are ubiquitous in blood vessels 30. Previous studies showed that KLF11 expression was inhibited after ischemic brain injury. Mounting evidence indicates that KLF11 overexpression protects against endothelial injury via mitigating inflammation, reducing TJ protein degradation and improving BBB impairment 15, 31. However, the significant role of KLFs in endothelial cells after SCI remains unclear. Recent studies have demonstrated that KLF2, KLF4, KLF5 and KLF6 are involved in regulating autophagy signaling pathways 17, 20, 32. Our results indicated that the KLF2, KLF4, KLF9, KLF14 and KLF16 mRNA levels were remarkably elevated after SCI.

Interestingly, both mRNA and protein expression of KLF2 in endothelial cells increased at 1 day after the injury. Given the ability of KLF2 to maintain vascular homeostasis, early induction of KLF2 by hypoxia may reflect an acutely protective effect of KLF2 against hypoxia and a potentially vasoprotective response. Reduced KLF2 expression may contribute to endothelial dysfunction and barrier disruption following SCI. Sergi and co-workers have recently demonstrated that autophagy promotes KLF2 expression and maintains endothelial phenotype and survival 33. KLF2 expression is correlated with the activity of autophagy, suggesting that KLF2 may play an essential role in regulating endothelial autophagy. Previous studies have indicated that KLF2 can regulate autophagy in two different cell development processes (i.e., osteoblast differentiation and osteoclastogenesis) with distinct molecular mechanisms 17, 18. In the present study, we found that overexpression of KLF2 significantly enhanced autophagosome activity and lysosomal function by promoting autophagosome fusion with the lysosome. These results were also confirmed in bEnd.3 cells transfected with Klf2 shRNA. As a transcription factor, KLF2 could bind to the promoter region of autophagy proteins to enhance their transcriptional activity 17, 18, 21.

Interestingly, we have found that KLF2 improves lysosomal function after autophagy activation. However, the detailed regulatory mechanism is still unclear. Past studies have shown that KLF2 directly binding to the TFEB promoter to activate TFEB mRNA transcription in endothelial cells 27. TFEB, as a member of the MiT family, is a master regulator of lysosome biogenesis and autophagy-related gene transcription. Our results suggested that TFEB was a key downstream of KLF2. We found that KLF2 overexpression activated TFEB and downregulated TFEB levels in OGD-treated cells bEnd.3 cells. Our results also revealed that abolished LV-KLF2 could enhance autophagy and lysosome function. Therefore, KLF2-induced autophagy-lysosome function in endothelial cells may be partly TFEB-dependent.

Our results showed that KLF2 overexpression inhibited the degradation of TJs and disruption of BSCB and promoted motor function recovery after SCI, whereas KLF2 downregulation reversed these promising effects. Consistent with previous reports 7, our findings revealed a protective role of KLF2-induced autophagy enhancement in maintaining BSCB integrity. Autophagy inhibits membranous ZO-1 into the cytosol. Meanwhile, autophagy participates in the degradation of delocalized ZO-1. Peter and colleagues have indicated that endothelial-derived KLF2 maintains blood flow and vasoreactivity and alleviates vascular inflammation and thrombosis 13. Although the current study demonstrated a critical role of KLF2 in BSCB regulation, we explored other mechanisms that may also contribute to outcomes following SCI. Previous studies have revealed that KLF2 deficiency promotes neuroinflammation and alleviates neurological dysfunction in multiple sclerosis mice 34. Furthermore, KLF2 improves regional cerebral blood flow after ischemic stroke by regulating endothelial synthase-derived nitric oxide 35. Therefore, further studies are required to explore the significant role of KLF2 in regulating blood flow and inflammatory response after SCI.

Previous studies confirmed that HMG CoA-reductase inhibitors (i.e., statins) might significantly promote KLF2 expression in endothelial cells 36. Recent studies have also suggested that statins promote the recovery of neurological function after SCI through multiple pathways 37. In addition, clinical studies showed that statin administration could reduce the mortality rate among a cohort of veterans with traumatic SCI 38. However, few studies have been conducted to explore the promising effects of statins modulating KLF2 expression and KLF2-associated function in endothelial cells. Therefore, combined with our experimental results, the protective effects of KLF2 on BSCB integrity after SCI may provide a potential therapeutic target for the treatment and management of SCI.

In summary, our study has revealed that SCI further induces lysosomal damage, disrupts autophagic flux and promotes ALP dysfunction in endothelial cells. Mechanistically, overexpression of KLF2 inhibited the degradation of TJ proteins, suppressed apoptosis and maintained endothelial barrier integrity by enhancing the ALP after SCI (Figure S9). Our results indicated that KLF2 played a regulatory role in ALP in endothelial cells and BSCB integrity after SCI. Therefore, our findings suggest that KLF2 could be a promising target to restore BSCB permeability for the treatment and management of SCI.

## Material and Methods

### Animals

All experiment protocols were approved by the Animal Research Committee of Wenzhou Medical University, China (wydw2022-0746). All animals utilized herein were treated as per the ethical guidelines on animal experimentation of the Laboratory Animals of China National Institutes of Health. Female C57BL/6 mice (Average weight 20~30g) were obtained from the Experimental Animal Center (License no. SCXK 2005-0019) of Wenzhou Medical University, Zhejiang Province, China.

### SCI model and group

C57BL/6 mice were randomly allocated into the Sham group, 1dpi group (1 day after SCI), 3dpi group (3 days after SCI) and 7dpi group (7 days after SCI); other mice were divided into SCI+3-MA, SCI+shRNA-Klf2 group, SCI+shRNA-Con group, SCI+LV-Klf2 group, SCI+LV-Con group and SCI+LV-Klf2+CQ group. Mice from the SCI+3-MA group and SCI+LV-Klf2+CQ group received daily intraperitoneal injections of 3-Methyladenine (3-MA, Sigma-Aldrich St. Louis, MO, USA, 15 mg/kg) and chloroquine (CQ, Sigma-Aldrich St. Louis, MO, USA, 50 mg/kg) for 3 days after SCI. The SCI contusion model was constructed according to the previous work 9. Briefly, after anesthesia with 0.3% sodium pentobarbital solution (0.2 ml/10g, ip), mice underwent a laminectomy at vertebral level T9-T10 to expose the dorsal cord surface without disrupting the dura. Subsequently, a 10g weight was dropped from 2.0 cm onto the exposed dorsal surface of the spinal cord to induce a moderate SCI model. Afterward, muscle and skin were sutured in layers. After the operation, the mice received daily manual urinary bladder emptying until the bladder reflex was re-established. The sham group mice were treated with anesthesia and a laminectomy but without SCI.

### Cell culture and oxygen-glucose deprivation (OGD) treatment

The bEnd.3 brain microvascular endothelial cell line was purchased from the Chinese Academy of Sciences (Shanghai, China). The cells were cultured in the DMEM containing 10% FBS and 1% penicillin-streptomycin at 37°C and 5% CO2. Cells were grown to 85% to 95% confluence before use.

For OGD induction, bEnd.3 cells were rinsed with glucose-free deoxygenated DMEM (Gibco, 11966025) and cultivated in a hypoxia chamber (Thermo Fisher Scientific, Waltham, Massachusetts, USA) maintained at 37°C for 3h, 6h, 9h and 12h respectively. The chamber was filled with premixed gas (5% CO2 and 95% N2). The controls were cultivated in DMEM and 10% FBS for the identical duration. For autophagy analysis, the cells were pretreated with 3-MA (10mM) or CQ (30 μM) for 2 h before the exposure to OGD.

### Lentiviral vector construction and transfection

For genetic manipulations of KLF2, the recombination lentiviruses, shRNA-Klf2, shRNA-Con, LV-Klf2 and LV-Con were established and synthesized by GeneChem Chemical Technology Co., Ltd. (Shanghai, China), according to the supplier's directions. Lentivirus vectors were produced (1 × 10^9^ transfection units (TU)/mL) and transfected in vivo via injecting intrathecally, as reported previously 39. After anesthesia, mice were placed on an operating surface that flexed their back. Once correct needle placement was verified, the solutions were injected within 1 min. Mice then put heads up for 30 min. Mice were included in the study only if they had normal hind-limb motility function 3 days after the intrathecal injection.

shRNA-Klf2, shRNA-Con, LV-Klf2 and LV-Con were also applied in vitro experiments. The adeno-associated virus (AAV)-TFEB shRNA and the control virus were formed and packaged by GeneChem Chemical Technology Co., Ltd. (Shanghai, China) and were also performed to knock down TFEB. The cells were subjected to lentivirus vector transfection at a confluence of 30-50%; >95% of the cells were viable 12 h later. The medium was then changed and the cells were cultivated for 3 days and passaged for additional assays.

### Western blot analysis

Spinal cord segments (10 mm length, epicenter in the middle) and cell specimens were dissected and processed by extracting proteins with a lysis buffer solution. The subcellular fractions of membrane, cytosol, and nuclear were obtained by using the membrane and cytosol protein extraction kit (Beyotime Institute of Biotechnology, Shanghai, China) or Nuclear and Cytoplasmic Protein Extraction Kit (Beyotime, China) respectively. Protein concentration was determined by BCA Protein Assay Kit (Beyotime, China), according to the supplier's instructions. Next, proteins were isolated via SDS-PAGE and moved to a PVDF membrane. After sufficient blocking with 10% non-fat dry milk solution for 2 h, the membranes were treated with specific primary antibodies at 4°C overnight. The membranes were then incubated with specific secondary antibodies (goat anti-mouse IgG (H+L) -HRP, Bioworld Technology Minneapolis, MN, USA, Cat. No. BS12478 and goat anti-rabbit IgG (H+L)-HRP, Bioworld Technology Minneapolis, MN, USA Cat. No. BS13278) for 1.5h at RT. The information about diluted concentrations of primary antibodies is indicated in Supplementary Table 1. B. The signal bands were visualized and quantified by Image Lab 3.0 software (Bio-Rad) via 'Torchlight' Ultrasensitive ECL Western HRP Substrate (Zen Bioscience, Chengdu, China).

### Immunofluorescence staining and confocal analysis

At 3 days after SCI, mice were anesthetized and euthanized by trans-cardiac perfusion with PBS followed by 4% (w/v) paraformaldehyde. The rostral spinal cord segments (1 mm in length, 4 mm far from the epicenter) were separated and post-fixed in PFA overnight. These specimens were sufficiently washed and embedded in paraffin. The slices (5μm) were mounted on gelatin-coated slides. The whole segments were prepared for longitudinal sections. Longitudinal slices of spinal cord segments were prepared for staining. Specimens were restored by high-pressure antigen retrieval after dewaxing and hydration. After adequate blocking with 5% bovine serum albumin (BSA) for 30 min at 37°C, the tissue section slides were incubated with specific primary antibodies at 4°C overnight. Then, the slides were re-incubated with secondary antibodies such as goat anti-rabbit IgG H&L (Alexa Fluor® 488) and goat anti-mouse IgG H&L (Alexa Fluor® TRITC) for 1h at room temperature and counterstained with DAPI reagent. The information on diluted concentrations of primary antibodies is listed in Supplementary Table 1. The images were captured using confocal microscopy (Nikon ECLIPSE 80i, Tokyo, Japan). The fluorescence intensity was then analyzed by ImageJ software (Fiji) and the integrated optical density (IOD) was calculated as (IOD = area × average density). To evaluate the expression level of TJ proteins, the stained signals in endothelial membrane or cytosol were selected and quantified respectively. The co-localization analysis (percentage of co-localization CTSD/LAMP1 and LC3/LAMP1) was assessed using the Coloc2 plugin in ImageJ software (Fiji) after individual thresholding frames.

### Monodansylcadaverine (MDC) staining

Cells were stained with MDC dye to measure the presence of autophagic vesicles (Sigma-Aldrich Chemical Company, Milwaukee, WI, USA, Cat. No. 10121-91-2) according to the manufacturer's instructions. Briefly, the cells were incubated with MDC (50 nM) at 37°C for 15 min. Then, the images were captured using confocal microscopy (Nikon ECLIPSE 80i, Tokyo, Japan). The mean fluorescence intensity was finally measured using ImageJ software (Fiji).

### Lyso-Tracker Red staining

The Lyso-Tracker Red staining (Beyotime Institute of Biotechnology, Shanghai, China, Cat. No. C1046) was performed to measure the presence of lysosomes, according to the supplier's instructions. The cells were incubated with Lyso-Tracker Red (50 nM) at 37°C for 30 min. Then, the images were captured using confocal microscopy (Nikon ECLIPSE 80i, Tokyo, Japan). The fluorescence intensity was finally measured using ImageJ software (Fiji).

### LC3 turnover assay

Autophagic flux was computed as the accumulation of autophagosomes after inhibition of autophagosomes-lysosomes fusion with CQ (100 μM) for 2h. For each experimental condition, autophagic flux = LC3-II(CQ) / GAPDH - LC3-II (vehicle) / GAPDH.

### Cell Viability

Cell viability was performed by Cell Counting Kit-8 (CCK-8, Beyotime Institute of Biotechnology, Shanghai, China, C0042). For CCK-8 assay, cells were seeded in 96-well plates at 5×10^3^ cells per well. To determine the effect of OGD on cell viability, cells were exposed to OGD for 3h, 6h, 9h and 12h respectively. To determine the effect of autophagy on cell viability, cells were pretreated with 3-MA (10mM) for 2 h before the exposure to OGD for 9h. After washing with PBS solution, 10 μL CCK-8 solution was added to each well and incubated with the cells at 37 °C for 4 h. The value of OD450 normalized to the control value was employed to reflect cell viability.

### Permeability assay

The extravasation of endothelial permeability was measured by quantifying FITC-dextran (70 kDa, Sigma Aldrich, USA). Cells were seeded in the 24-well transwell chambers with 0.4-mm pores (Corning, USA) and reached confluence. 100 μl of FITC-dextran medium (10 mg/mL) was added to the apical chamber and 500 μl of the medium was added to the basal chamber. After incubation for 4 h, the medium in the basal chamber was harvested for fluorescence measurement using an EnSpire Manager (PerkinElmer Company, USA) multimode microplate reader.

### Assessment of BSCB permeability

The BSCB permeability was evaluated by Evans blue (EB, Sigma-Aldrich St. Louis, MO, USA, Cat. No. 314-13-6) dye extravasation method as previously 40. EB dye (2% in saline, 200 μl) was employed on day 3 after SCI via i.v. 2 h before the mice execution. After anesthesia, mice were euthanized and received trans-cardiac perfusion with phosphate-buffered saline (PBS). The spinal cord lesion was removed and weighed for qualitative measurement of EB dye extravasation. Following incubation with N, N-dimethyl formamide (Sigma-Aldrich St. Louis, MO, USA) for 3 days at 72 °C and centrifugation for 45 min at 15,000 rpm, cell supernatants were harvested and the fluorescence signals were quantified using a spectrophotometer at 610 nm excitation and 680 nm emission wavelengths. The EB dye content was quantitatively calculated using a standard curve.

### Behavioral assessment

#### Basso mouse scale (BMS) behavioral analysis

The recovery of hind-limb motility function was performed with the BMS by measuring hind-limb joint activities, trunk position and stability, front and rear limbs coordination, paw placement, toe clearance and tail position. The scores were recorded before surgery and at 1, 3, 7, 14, 21 and 28 days after SCI. Mice were placed in an open field and the activities were observed and recorded by two investigators blinded to the treatment group.

#### Footprint analysis

Footprint analysis was performed to assess gait and motor coordination at 28 days after SCI. The front and hind paws of mice were stained with blue and red dyes, respectively. The mice were then encouraged to walk straight on a paper-lined runway. The stride lengths were measured and analyzed only when the mice ran at a constant velocity.

#### Electrophysiology assay

Electrophysiology assay was performed to examine motor-evoked potentials (MEPs) at 28 days after SCI. After anesthesia, an electrode was placed on the rostral ends of the exposed spinal cord. Then, a recording electrode was inserted into the biceps femoris flexor cruris (1.5 mm deep). Next, a reference electrode was placed at the distal tendon of the hind-limb muscle and the grounding line was placed subcutaneously. A single square wave (0.5mA, 0.5ms, 1Hz) was applied to induce MEPs and peak-to-peak amplitudes were calculated to assess the function of limb nerve conduction.

### Quantitative real-time polymerase chain reaction (RT-PCR)

Total RNA was extracted from the spinal cord lesion using the High Pure RNA Isolation Kit. According to the standard protocol of PrimeScript™ RT Master Mix (Takara Biomedical Technology, Japan, RR036A), cDNA was synthesized by reverse transcriptase. The qPCR assay was carried out using TB Green Master Reagents (Takara Biomedical Technology, Japan, RR820A). The amplification parameters were as follows: denaturation at 95°C for 30 s, annealing at 95°C for 5 s and extension at 60°C for 34 s for 40 cycles and the signal was detected at 60°C. Finally, the target mRNAs were normalized to GAPDH mRNA. The information about primers is listed in Supplementary Table 2.

### Statistical analysis

Statistical assays were performed using SPSS 19 (Chicago, IL, USA). All data were displayed as mean ± standard error of the mean (SEM). Unpaired Student's t-tests were performed for the significant difference in the two groups. One-way ANOVA with LSD (equal variances assumed) post-hoc analysis or Dunnett's T3 (equal variances not assumed) method was performed to evaluate significant differences between two groups in three or four groups. The BMS was analyzed via two-way ANOVA followed by the Bonferroni post-hoc test. P <0.05 had significance on statistics.

## Supplementary Material

Supplementary figures and tables.
