# Supplementary material for: Kruppel-like factor 2 contributes to blood-spinal cord barrier integrity and functional recovery from spinal cord injury by augmenting autophagic flux
Source: Theranostics. 2023 Jan 1;13(2):849–66. doi: 10.7150/thno.74324 (PMC9830435; doi:10.7150/thno.74324)
Supplement: Supplementary file 1 — Supplementary figures and tables. [file thnov13p0849s1.pdf]

Supplemental Data

Kruppel-like factor 2 contributes to blood-spinal cord barrier integrity and functional recovery from spinal cord injury by augmenting autophagic flux

Zili He [1,2#], Jiqing Du [2#], Yu Zhang [2], Yitie Xu [2], Qian Huang [2], Qingwei Zhou [2], Min Wu [2], Yao Li [1], Xie Zhang [3], Hongyu Zhang [2], Yuepiao Cai [1,2], Keyong Ye [2], Xiangyang Wang [1], Yingze Zhang [1,4], Qi Han [5], Jian Xiao [1,2]

1 Oujiang Laboratory (Zhejiang Lab for Regenerative Medicine, Vision and Brain Health), Department of Orthopaedics Surgery, The Second Affiliated Hospital and Yuying Children's Hospital of Wenzhou Medical University, Wenzhou, 325035, China.

2 Department of orthopaedics, Affiliated Pingyang Hospital and School of Pharmaceutical Science, Wenzhou Medical University, Wenzhou, 325000, China.

3 Department of Pharmacy, Ningbo Medical Treatment Center Li Huili Hospital, Ningbo, 315040, China

4. Department of Orthopaedic Surgery, The Third Hospital of Hebei Medical University, Shijiazhuang, 050051 China

5 Department of Anatomy and Physiology, Shanghai Jiao Tong University School of Medicine, Shanghai, 200025, China.

# These authors contributed equally to this work.

Correspondence should be addressed to Jian Xiao: xfxj2000@126.com, Qi Han: qihan8@shsmu.edu.cn, and Yingze Zhang: yzling_liu@163.com

Supplemental Figure legends

Figure S1 Disruption of autophagy exacerbates endothelial apoptosis level.

(A) Cell viability in bEnd.3 cells exposed to OGD for 0, 3, 6, 9 and 12 h. n = 4. (B) Effect of OGD (9 h) on protein expression of LC3 and p62 from bEnd.3 cells pretreated in the absence or presence of 3-MA. n = 4 sample. (C) Effect of OGD (9 h) on protein expression of apoptosis markers (Bcl-2, Bax and Cleaved caspase 3) from bEnd.3 cells pretreated in the absence or presence of 3-MA. n = 3 sample. (D-G) Representative immunofluorescence images and quantification of LC3 (E), p62 (F), and Caspase 3 (C) in bEnd.3 cells. n = 3 sample. Shown are mean values ± SEM; ns stands for not significant, * $P < 0.05$, ** $P < 0.01$.

Figure S2 Disruption of autophagy exacerbates ZO-1 degradation after SCI.

(A) Western blotting and quantification of autophagy markers (LC3 and p62) in spinal cord tissue prepared from SCI (3dpi) and SCI + 3-MA group. n = 6 mice in each group. (B-C) Representative immunofluorescence images and quantification of LC3 (B) and P62 (C) in endothelial cells (marked by CD31) in indicated groups at day 3 after injury.

n = 5 mice in each group. Shown are mean values ± SEM; ns stands for not significant, * $P < 0.05$, ** $P < 0.01$.

Figure S3 The expression of KLF2 in bEnd.3 cells following OGD.

(A) Western blotting and quantification of KLF2 in bEnd.3 cells after OGD treatment for different periods. n = 4 sample. (B) Representative immunofluorescence images and quantification of KLF2 in bEnd.3 cells. n = 3. Shown are mean values ± SEM; ns stands for not significant, * $P < 0.05$, ** $P < 0.01$.

Figure S4 The overexpression and knockdown efficiency of cell transfection.

bEnd.3 cells were respectively transfected with lentivirus-Klf2 or lentivirus-Klf2 shRNA to overexpress or knockdown KLF2 expression. (A-B) Western blotting and quantification of KLF2 in bEnd.3 respectively transfected with lentivirus-Klf2 (A) or lentivirus-Klf2 shRNA (B). (C-D) Western blotting and quantification of p62 expression in bEnd.3 respectively transfected with lentivirus-Klf2 (C) or lentivirus-Klf2 shRNA (D). Shown are mean values ± SEM; ns stands for not significant, * $P < 0.05$, ** $P < 0.01$.

Figure S5 OGD-induced cell apoptosis is alleviated by KLF2 overexpression in bEnd.3 cells.

(A) Western blotting and quantification of KLF2 in bEnd.3 cells respectively transfected with LV-Con or LV-Klf2, and pretreated with OGD treatment. n = 3 sample.

(B) Effect of OGD (9 h) on protein expression of apoptosis markers (Bcl-2, Bax and Cleaved caspase 3) from bEnd.3 cells respectively transfected with LV-Con or LV-Klf2. n = 4 sample. (C) Effect of OGD (9 h) on protein expression of TJs markers (ZO-1, OCC and claudin-5) from bEnd.3 cells respectively transfected with LV-Con or LV-Klf2, and pretreated in the absence or presence of CQ. n = 4 sample. (D) Representative immunofluorescence images and quantification of Caspase 3 in bEnd.3 cells. n = 3 sample. Shown are mean values ± SEM; ns stands for not significant, * $P < 0.05$, ** $P < 0.01$.

Figure S6 Schematic of experimental timeline and Lentivirus injection.

(A)Experimental protocol. We transfected mice with lentiviral vectors (LV-Klf2 or shRNA-Klf2) via intrathecal injection 5 days prior to SCI to overexpress or knockdown KLF2. (B) The expressions of LC3II and p62 in mice respectively transfected with LV-Con or LV-Klf2. n = 5 mice in each group. (C-D) The Western blot analysis showed that the expressions of KLF2 were overexpressed (C) or knock downed (D) in the spinal cord at day 3 after injury. n = 3 mice in each group. Shown are mean values ± SEM; ns stands for not significant, * $P < 0.05$, ** $P < 0.01$.

Figure S7 TFEB regulated KLF2 mediated ALP in in bEnd.3 cells exposed to OGD.

bEnd.3 cells were treated with Ad-shTFEB for 24 h and then with LV-KLF2, and then exposed to OGD for 9 h; (A) Western blot and quantification of TFEB in nuclear

subfractions of OGD-treated bEnd.3 cells. n = 4 sample. (B) Western blot and quantification of autophagy lysosome pathway markers (LC3, p62 and LAMP2) in bEnd.3 cells respectively transfected with Ad-shTFEB and LV-Klf2 after OGD treatment (9 h). n = 4 samples. (C-D) Expression of mRNA encoding TFEB (C) and protein expression of TFEB (D) in bEnd.3 cells transfected with LV-Klf2. n = 4 samples. (E) Relative mRNA level of TFEB in bEnd.3 cells transfected with shRNA-Klf2. n = 4 samples. (F) Representative immunofluorescence images and quantification of TFEB in bEnd.3 cells transfected with LV-Klf2. n = 3 sample. Shown are mean values ± SEM; ns stands for not significant, * $P < 0.05$, ** $P < 0.01$.

Figure S8 KLF2 ameliorates BSCB disruption and motor function impairment in SCI.

(A) Western blot and quantification of TJs markers (ZO-1, OCC and claudin-5) in spinal cord tissue from Sham, SCI, SCI+LV-Con, SCI+LV-Klf2, and SCI+LV-Klf2+CQ, group at day 3 after injury. n = 5 mice in each group. (B-C) Representative MEPs and amplitude analysis of mice at day 28 after SCI. n = 5 mice in each group. (D) BMS score analysis in each group at the indicated time points. n = 7 mice in each group. (E) Representative foot printing analysis of mice at day 28 after injury. (F) Quantification of stride lengths of hind footprints of mice. n = 5 mice in each group. Shown are mean values ± SEM; ns stands for not significant, * $P < 0.05$, ** $P < 0.01$.

Figure S9 Proposed model of how KLF2 contributes to BSCB integrity in SCI.

SCI induced the ALP dysfunction in endothelial cells disturbs redistribution of membranous TJ proteins and clearance of delocalized TJ proteins which impairs the integrity and permeability of BSCB. Meanwhile, endothelial KLF2 was suppressed after SCI, upregulated of which enhances autophagy levels and lysosomal function via regulating TFEB expression to suppress redistribution of membranous TJ proteins and to further reduce cytotoxicity, attenuating BSCB disruption.

Figure S1

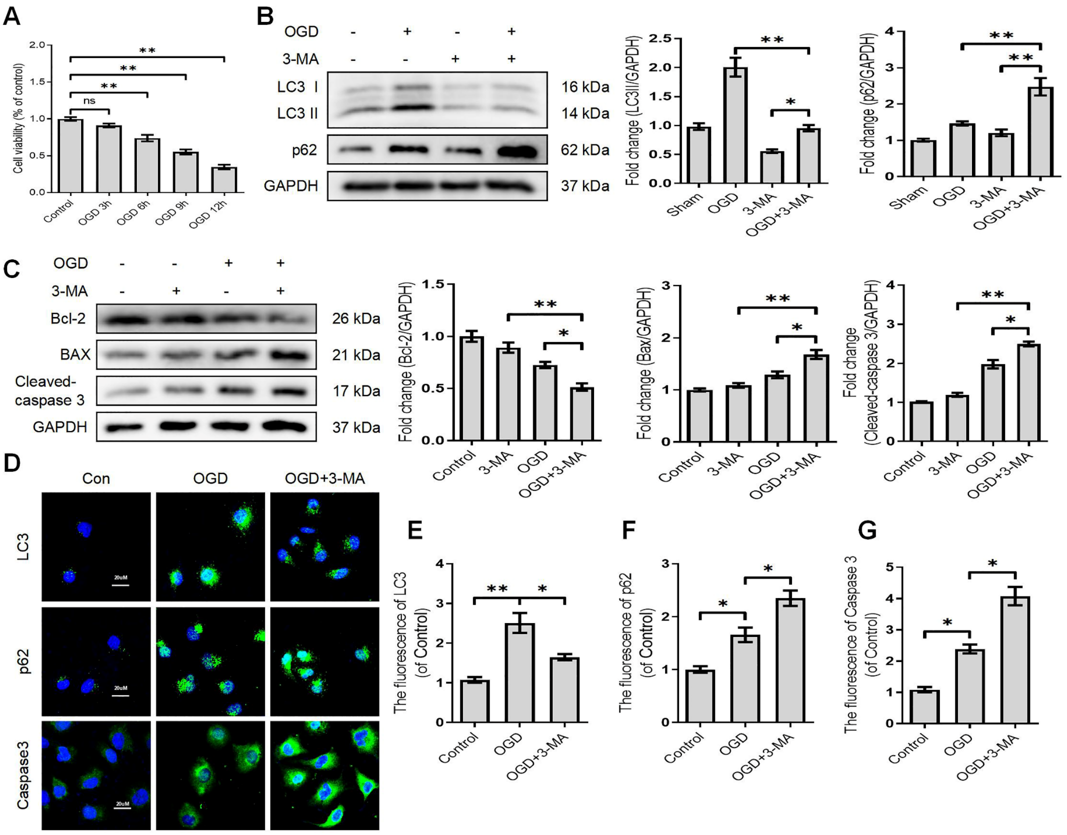
A
B
OGD
3-MA
LC3 I
LC3 II
p62
GAPDH
16 kDa
14 kDa
62 kDa
37 kDa
Fold change (LC3II/GAPDH)
Fold change (p62/GAPDH)
Sham
OGD
3-MA
OGD+3-MA
Cell viability (% of control)
Control
OGD 3h
OGD 6h
OGD 9h
OGD 12h
C
Bcl-2
BAX
Cleaved-caspase 3
26 kDa
21 kDa
17 kDa
Fold change (Bcl-2/GAPDH)
Fold change (Bax/GAPDH)
Fold change (Cleaved-caspase 3/GAPDH)
D
Con
OGD+3-MA
LC3
p62
Caspase3
E
F
G
The fluorescence of LC3 (of Control)
The fluorescence of p62 (of Control)
The fluorescence of Caspase 3 (of Control)

Figure S2

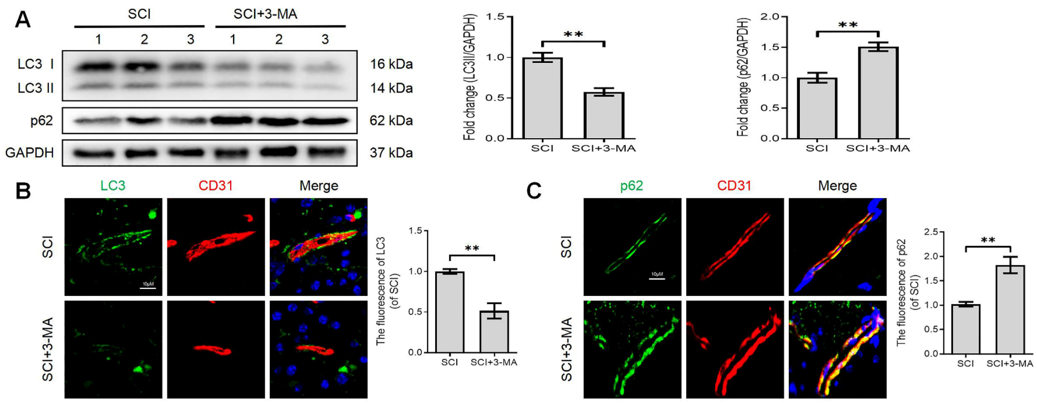
A
SCI
SCI+3-MA
1
2
3
1
2
3
LC3 I
LC3 II
p62
GAPDH
16 kDa
14 kDa
62 kDa
37 kDa
Fold change (LC3II/GAPDH)
SCI
SCI+3-MA
Fold change (p62/GAPDH)
SCI
SCI+3-MA
B
LC3
CD31
Merge
SCI
SCI+3-MA
The fluorescence of LC3 (of SCI)
SCI
SCI+3-MA
C
p62
CD31
Merge
SCI
SCI+3-MA
The fluorescence of p62 (of SCI)
SCI
SCI+3-MA

FigureS3

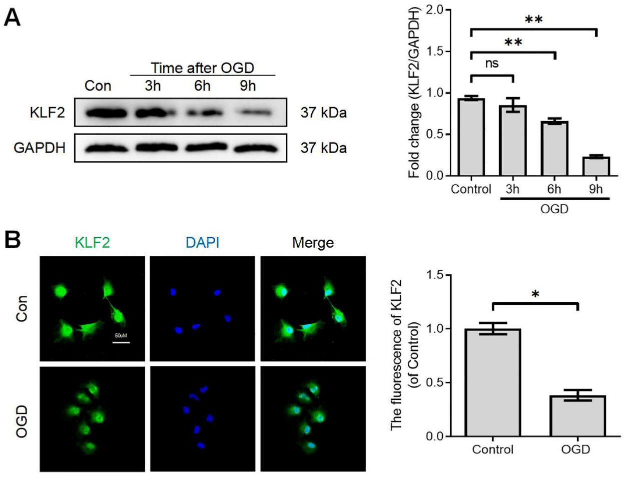
A
Time after OGD
Con
3h
6h
9h
KLF2
GAPDH
37 kDa
37 kDa
Fold change (KLF2/GAPDH)
2.0
1.5
1.0
0.5
0.0
**
**
ns
Control
3h
6h
9h
OGD
B
KLF2
DAPI
Merge
Con
OGD
The fluorescence of KLF2 (of Control)
1.5
1.0
0.5
0.0
*
Control
OGD

Figure S4

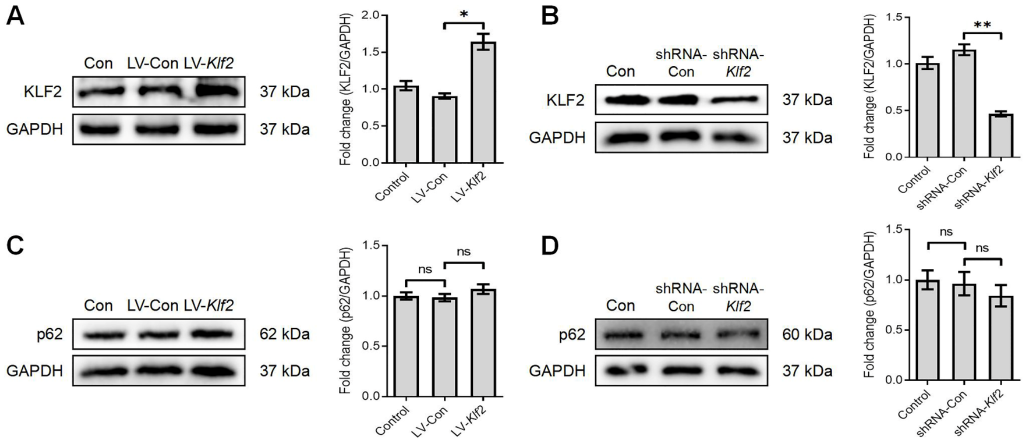
A
Con
LV-Con
LV-Klf2
KLF2
37 kDa
GAPDH
37 kDa
Fold change (KLF2/GAPDH)
2.0
1.5
1.0
0.5
0.0
*
Control
LV-Con
LV-Klf2
B
Con
shRNA-Con
shRNA-Klf2
KLF2
37 kDa
GAPDH
37 kDa
Fold change (KLF2/GAPDH)
1.5
1.0
0.5
0.0
**
Control
shRNA-Con
shRNA-Klf2
C
Con
LV-Con
LV-Klf2
p62
62 kDa
GAPDH
37 kDa
Fold change (p62/GAPDH)
1.5
1.0
0.5
0.0
ns
ns
Control
LV-Con
LV-Klf2
D
Con
shRNA-Con
shRNA-Klf2
p62
60 kDa
GAPDH
37 kDa
Fold change (p62/GAPDH)
1.5
1.0
0.5
0.0
ns
ns
Control
shRNA-Con
shRNA-Klf2

Figure S5

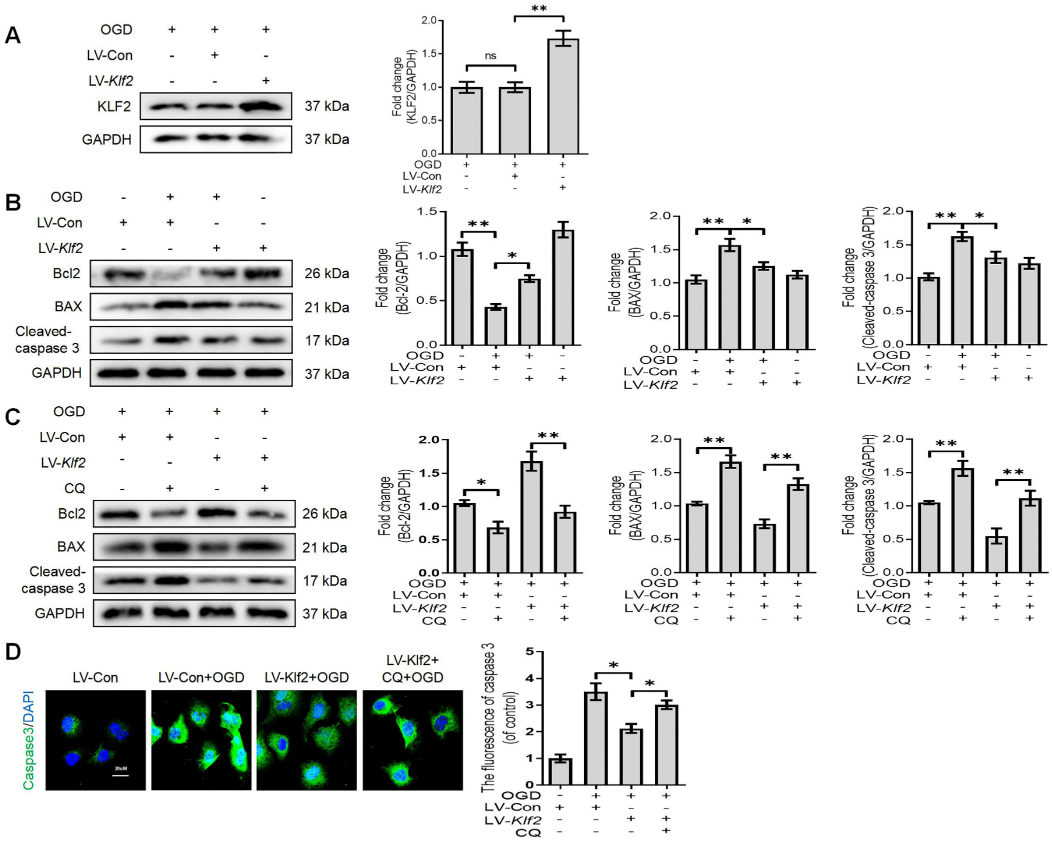
A
OGD
LV-Con
LV-Klf2
KLF2
37 kDa
GAPDH
37 kDa
Fold change (KLF2/GAPDH)
ns
**
B
OGD
LV-Con
LV-Klf2
Bcl2
26 kDa
BAX
21 kDa
Cleaved-caspase 3
17 kDa
GAPDH
37 kDa
Fold change (Bcl-2/GAPDH)
Fold change (BAX/GAPDH)
Fold change (Cleaved-caspase 3/GAPDH)
C
OGD
LV-Con
LV-Klf2
CQ
Bcl2
26 kDa
BAX
21 kDa
Cleaved-caspase 3
17 kDa
GAPDH
37 kDa
Fold change (Bcl-2/GAPDH)
Fold change (BAX/GAPDH)
Fold change (Cleaved-caspase 3/GAPDH)
D
LV-Con
LV-Con+OGD
LV-Klf2+OGD
LV-Klf2+CQ+OGD
Caspase3/DAPI
The fluorescence of caspase 3 (of control)

Figure S6

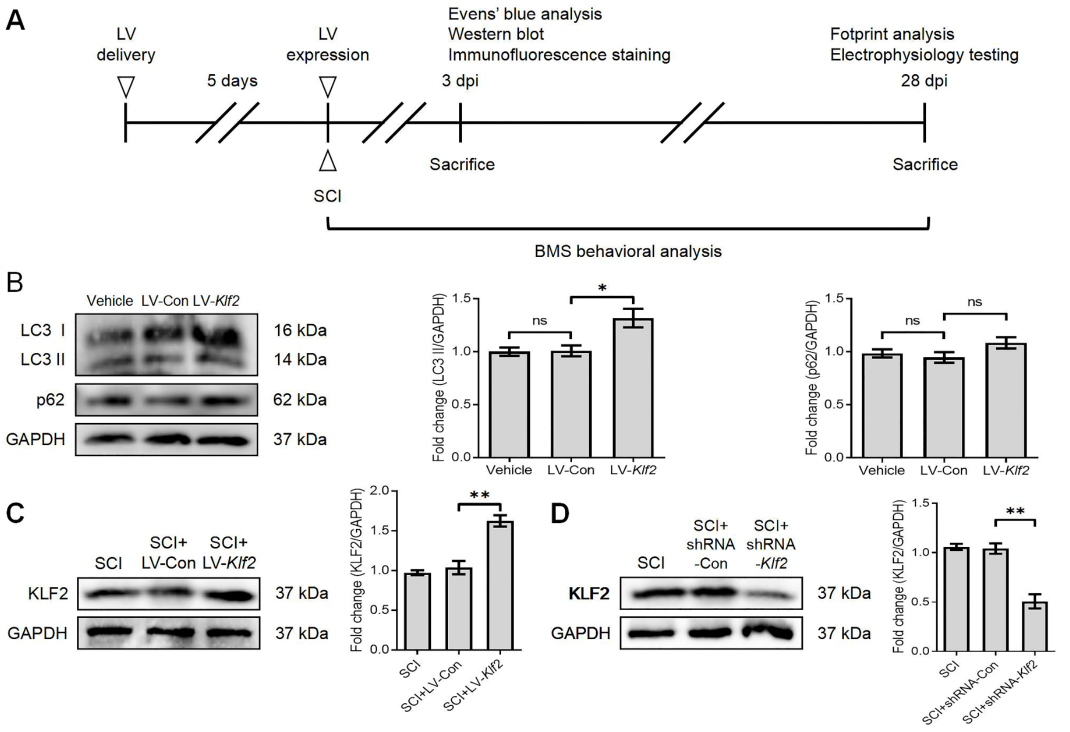

A
LV delivery
5 days
LV expression
Evens' blue analysis
Western blot
Immunofluorescence staining
3 dpi
Fotprint analysis
Electrophysiology testing
28 dpi
Sacrifice
SCI
Sacrifice
BMS behavioral analysis
B
Vehicle LV-Con LV-Klf2
LC3 I
LC3 II
p62
GAPDH
16 kDa
14 kDa
62 kDa
37 kDa
Fold change (LC3 II/GAPDH)
Fold change (p62/GAPDH)
ns
*
Vehicle LV-Con LV-Klf2
C
SCI SCI+ LV-Con SCI+ LV-Klf2
KLF2
GAPDH
37 kDa
Fold change (KLF2/GAPDH)
**
SCI SCI+LV-Con SCI+LV-Klf2
D
SCI SCI+ shRNA -Con SCI+ shRNA -Klf2
KLF2
GAPDH
37 kDa
Fold change (KLF2/GAPDH)
SCI SCI+shRNA-Con SCI+shRNA-Klf2

129 **Figure S7**

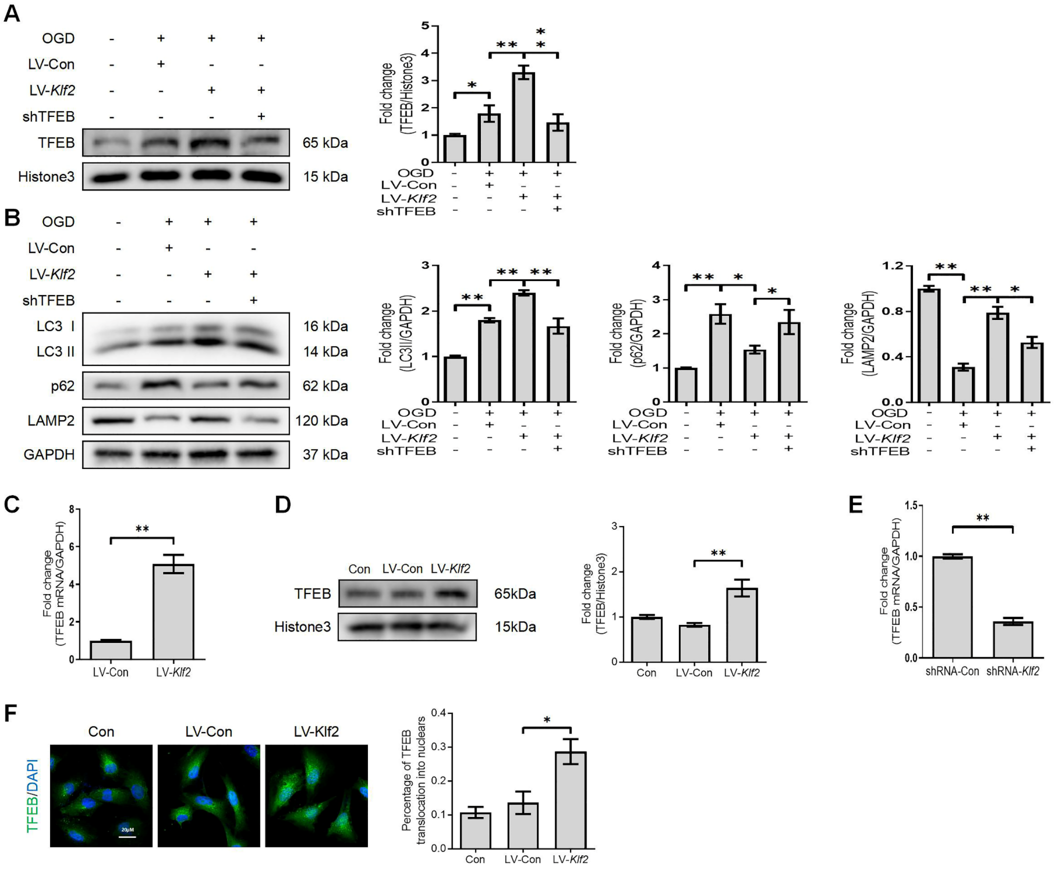

130

Figure S8

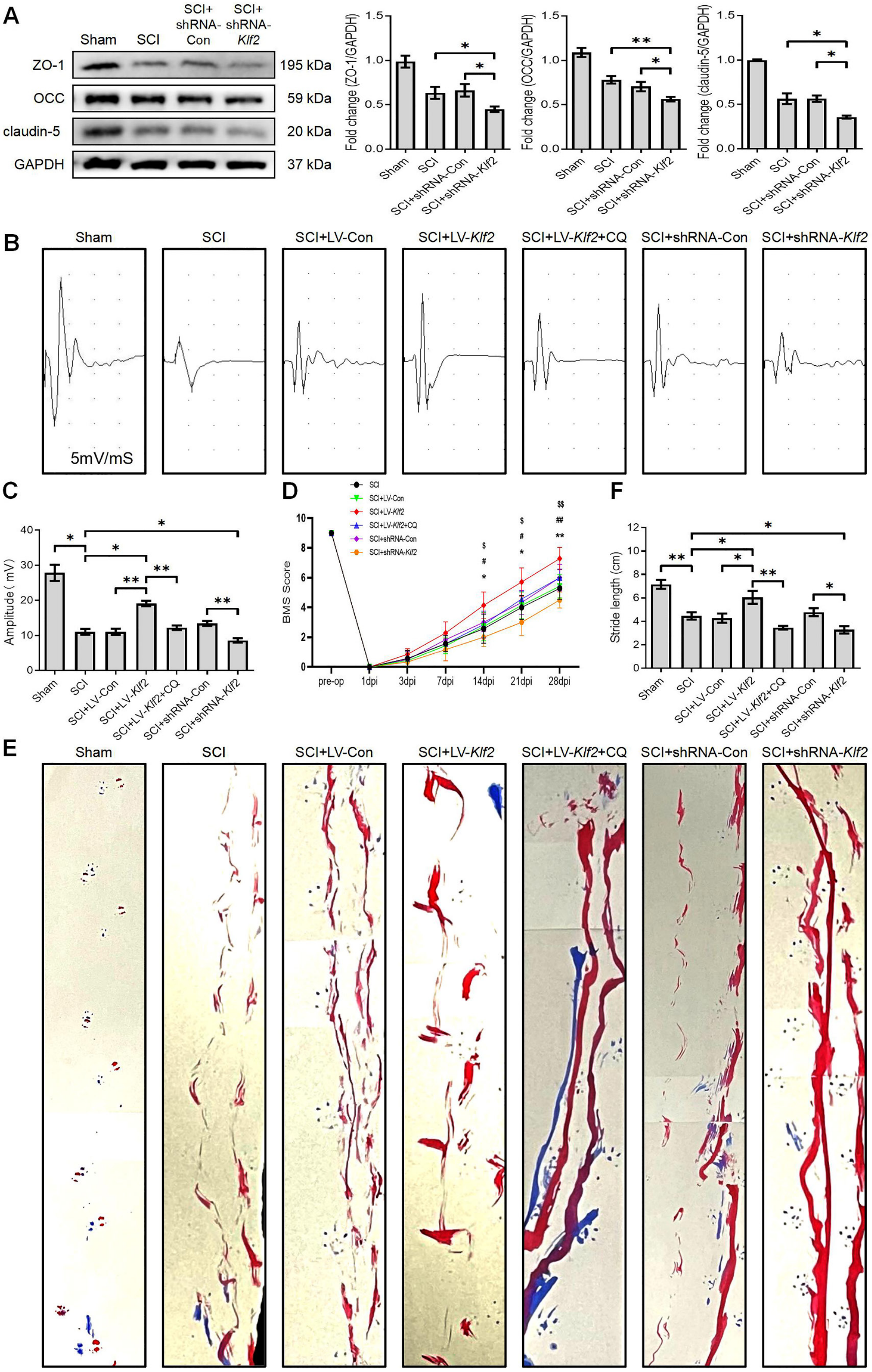

A
Sham
SCI
SCI+ shRNA-Con
SCI+ shRNA-Klf2
ZO-1
OCC
claudin-5
GAPDH
195 kDa
59 kDa
20 kDa
37 kDa
Fold change (ZO-1/GAPDH)
Fold change (OCC/GAPDH)
Fold change (claudin-5/GAPDH)
SCI+shRNA-Con
SCI+shRNA-Klf2
B
SCI+LV-Con
SCI+LV-Klf2
SCI+LV-Klf2+CQ
5mV/mS
C
Amplitude (mV)
D
BMS Score
pre-op
1dpi
3dpi
7dpi
14dpi
21dpi
28dpi
F
Stride length (cm)
E

Figure S9

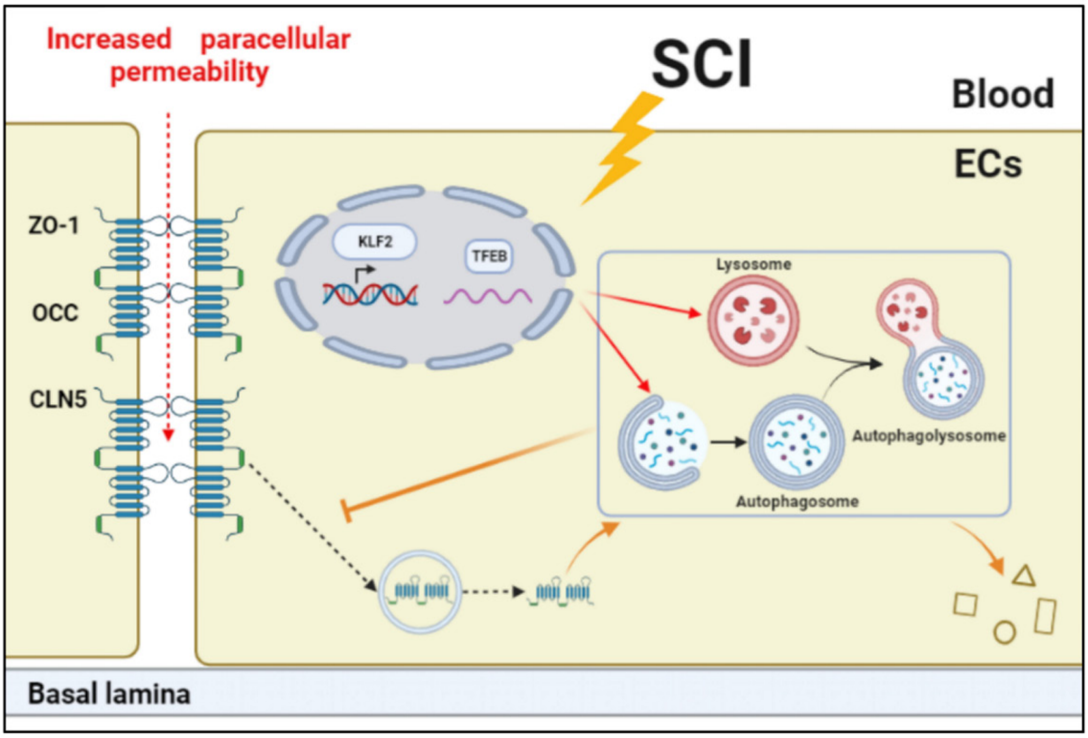

Increased paracellular permeability
SCI
Blood
ECs
ZO-1
OCC
CLN5
KLF2
TFEB
Lysosome
Autophagolysosome
Autophagosome
Basal lamina

Table S1

Table S1. The detail information of primary antibodies.

**Antibodies**	**Cat. No.**	**Company**	**Concentration for WB**	**Concentration for IF**
KLF2	DF13602	Affinity (Cincinnati, OH, USA)	1:1000	1:100
CD31	sc-376764	Santa Cruz Biotechnology (CA, USA)	/	1:50
claudin 5	343214	Zen bioscience (Chengdu, China)	1:1000	/
OCC	13409-1-AP	Proteintech Group (Chicago, IL, USA)	1:1000	1:200
ZO-1	AF5145	Affinity (Cincinnati, OH, USA)	1:1000	1:200
LC3	14600-1-AP	Proteintech Group (Chicago, IL, USA)	1:1000	1:200
P62/SQSTM1	18420-1-AP	Proteintech Group (Chicago, IL, USA)	1:1000	/
Beclin 1	11306-1-AP	Proteintech Group (Chicago, IL, USA)	1:1000	/
CTSD	380946	zen-bioscience (Chengdu, China)	1:1000	1:200
LAMP1	sc-20011	Santa Cruz Biotechnology (CA, USA)	/	1:100
LAMP2	27823-1-AP	Proteintech Group (Chicago, IL, USA)	1:1000	1:100
Caspase 3	19677-1-AP	Proteintech Group (Chicago, IL, USA)	1:1000	1:200
Bcl2	26593-1-AP	Proteintech Group (Chicago, IL, USA)	1:1000	/
BAX	50599-2-Ig	Proteintech Group (Chicago, IL, USA)	1:1000	/
TFEB	PA5-96632	Thermo Scientific (Madison, WI, USA)	1:1000	1:200
ATP1A1	GB11400	Servicebio (Wuhan,China)	1:500	/
Histone 3	384572	zen-bioscience (Chengdu, China)	1:1000	/
GAPDH	380626	zen-bioscience (Chengdu, China)	1:5000	/

Table S2

Table S2. The detail information of primers.

Primers	Forward primer	Reverse primer
Klf1	TCTGAGGAGACGCAGGATTTG	ACAGGTCACGTCCCTCTCATC
Klf2	GAGCCTATCTTGCCGTCCTTT	CACGTTGTTTAGGTCCTCATCC
Klf3	GATGAAGCCCAACAAATATGGGG	TCCACCTGTATCCCGTGAGTG
Klf4	AGGAACTCTCTCACATGAAGCG	GGTCGTTGAACTCCTCGGTC
Klf5	CAGGCCACCTACTTTCCCC	GAATCGCCAGTTTGGAAGCAA
Klf6	GTTTCTGCTCGGACTCCTGAT	TTCCTGGAAGATGCTACACATTG
Klf7	AGTGGACATTTTGCTCTCTCG	GTTAATGAGGTCACTGCGTTGA
Klf8	CTGGAGAGTGATTTCAACATGCC	GGAGGACGGATTGGAGCTT
Klf9	GCCGCCTACATGGACTTCG	GGTCACCGTGTTCCTTGGT
Klf10	ATGCTCAACTTCGGCGCTT	CGCTTCCACCGCTTCAAAG
Klf11	CCCCACTCAAGAGCAACGAG	CCAAGTTAGTGACGAGTAAGCC
Klf12	GTCAAAACCGAGCTTGTGGAA	GGGCTCCCCTTTCACATTATTT
Klf13	CCTGGCCTCAGACAAAGGG	ATTTCCCGTAAACTTTCTCGCA
Klf14	CTCCGTGTGCCTCAACTAGC	CAGGCGCATCCAGGATAGC
Klf15	CAGAGAGCGTCAAGGTCGC	TTCGCACAAACTTTGAGGGCA
Klf16	AGCATCCTGGCCGATCTGA	GTGCGAAGACTTGTAATAGGCT
Klf17	AATAAGGAACAGGCTATGCACC	GTGGCTGATGAAATCCGCTG
Map1lc3b	TTATAGAGCGATACAAGGGGGAG	CGCCGTCTGATTATCTTGATGAG
Sqstm1/p62	GAACTCGCTATAAGTGCAGTGT	AGAGAAGCTATCAGAGAGGTGG
TFEB	ACAAGGCACCATCCTCA	CCAGCTCGGCCATATTCA
Gapdh	AGGTCGGTGTGAACGGATTTG	GGGGTCGTTGATGGCAACA
